# Weather conditions structure the taxonomic and functional diversity of the aeolian dust microbiome

**DOI:** 10.3389/fmicb.2026.1691133

**Published:** 2026-03-25

**Authors:** Linton Freund, Talyssa M. Topacio, Yaning Miao, William C. Porter, Mark Swenson, Mia Maltz, Jon Botthoff, Emma L. Aronson

**Affiliations:** 1 Genetics, Genomics, and Bioinformatics Program, University of California, Riverside, Riverside, CA, United States; 2 Department of Microbiology and Plant Pathology, University of California, Riverside, Riverside, CA, United States; 3 Department of Environmental Sciences, University of California, Riverside, Riverside, CA, United States; 4 Department of Entomology, University of California, Riverside, Riverside, CA, United States; 5 Department of Plant Science and Landscape Architecture, University of Connecticut, Storrs, CT, United States; 6 Center for Conservation Biology, University of California, Riverside, Riverside, CA, United States

**Keywords:** aeolian microbiome, dust microbiome, functional diversity, metagenomes, microbial ecology, Salton Sea

## Abstract

**Introduction:**

The aeolian dust microbiome is composed of uniquely adapted microorganisms that can withstand the harsh conditions of the atmosphere. Specific microbial taxa and survival strategies have been observed in dust microbiomes from around the world, yet the environmental processes that select for microbial composition and function are poorly understood.

**Methods:**

Here we explore the taxonomic and functional diversity of the aeolian dust microbiome from sites around the Salton Sea, a hypersaline lake in Southern California, and how dust sources and weather influenced the microbiome. Dust samples were collected from four locations around the Salton Sea in the summer and fall of 2020 and 2021, and 16S (V3–V4) rRNA amplicon sequencing and shotgun metagenomic sequencing was used to characterize the aeolian dust microbiome.

**Results:**

We observed significant differences in microbial composition between sites, and we were able to identify 13 microbial genera that were members of the core dust microbiome across samples. We also found that genes involved in sporulation, UV-radiation resistance, thermal resistance, osmotic stress resistance, quorum sensing, and antibiotic resistance were shared across the aeolian dust metagenomes. Lastly, local wind conditions and estimated dust source surface categories were significant predictors of the microbial adaptations we found in the aeolian dust metagenomes.

**Discussion:**

Our results demonstrate the ability of airborne dust microorganisms to readily adapt to their harsh environment and highlight the survival mechanisms that allow them to disperse across broad distances, thus posing a potential health risk to exposed communities.

## Introduction

Microorganisms are ubiquitous and can be found in every environment, even in the most arduous and extreme ecosystems. Aeolian (i.e., windblown) dust is an example of such a system and is garnering more attention recently by microbial ecologists. This is in part because global dust load has increased by around 55% since the mid-1800’s (Kok et al., 2018), impacting local and global climate, as well as public health. Dust is responsible for delivering abiotic and biotic particulates across thousands of kilometers, contributing to aerosolized particulate matter (PM) load in the atmosphere. Microorganisms become entrained in dust either as free-floating single cells or spores, or by attaching to existing particulates or aerosols to form aggregates, which can then serve as protection as they travel (Erkorkmaz et al., 2023). Some microorganisms can live in the atmospheric environment, while others are readily dispersed and subsequently introduced to new ecosystems (Maltz et al., 2022). Like other forms of particulate matter, aeolian dust microorganisms effect the weather by acting as cloud or ice nucleating agents (Amato et al., 2015; Joly et al., 2015), contributing to the formation of clouds and affecting Earth’s overall radiative budget (Kok et al., 2018; Shi and Liu, 2019). They can also contribute to changes in the local ecology via dispersal and deposition, influencing the nutrient cycling dynamics in these new habitats. Furthermore, these microorganisms can also negatively impact public health as they can travel long distances and enter the respiratory tract via inhalation, leading to respiratory distress and lung microbial dysbiosis (Mayol et al., 2014; Hufnagl et al., 2020; Biddle et al., 2021; Maltz et al., 2022, 2025). The assembly of the microbial communities associated with airborne dust is of crucial importance due to its implications for both ecological and human health, demonstrating the strong connections between this ubiquitous microbiome, our own 81 health, and the health of our environment.

For microorganisms to survive in aeolian dust they must overcome environmental stressors that are not commonly experienced in terrestrial or aquatic ecosystems. First, microorganisms can be introduced into the atmosphere via dust emission or entrainment and then are subjected to wind shear stress in the process. Once at higher altitudes, these microorganisms can be exposed to UV-radiation from unobstructed sun exposure and experience rapid changes in temperature and pressure as the wind travels both horizontally and vertically through atmospheric advection (Schepanski, 2018). Additionally, moisture and nutrient availability is especially unpredictable in aeolian dust, which can lead to osmotic stress, desiccation stress, and starvation (Tang et al., 2018). Thus, the aeolian dust microbiome must contain the adaptations required to survive these unique atmospheric stressors. Airborne microbial communities sampled around the world have been found to contain genes involved in UV-radiation resistance, osmotic stress resistance, and thermal resistance (Aalismail et al., 2019). Some Gram-positive dust microorganisms have been found to form endospores, allowing them to endure the fluctuating conditions of the atmosphere (Bragoszewska et al., 2017). Lastly, both Gram-positive and Gram-negative bacteria can form biofilms, which may assist them in adhering to dust particulates in the air (Hu et al., 2024). This allows the bacteria to form aggregates which can shield them against wind shear stress and provide nutrition when nutrients are scarce. Collectively, the combined environmental pressures experienced by aeolian dust characteristically selects for these adaptations in dust microbiomes.

The Salton Sea ecosystem in Southern California presents a unique and urgent opportunity to explore the mechanisms that structure the taxonomic and functional composition of its aeolian dust microbiome. The Salton Sea is a hypersaline lake that is rapidly shrinking, exposing its playa sediment to wind erosion and thus increasing the dust load in the atmosphere. Emissions from increasingly dried lakebed surfaces have been associated with increases in PM in the area (Parajuli and Zender, 2018; Jones and Fleck, 2020). These dust emissions have also been implicated in local increases in respiratory illnesses (Farzan et al., 2019; Jones and Fleck, 2020) and have been shown to induce lung inflammation upon exposure in a lab setting (Biddle et al., 2021, 2023; Maltz et al., 2025). Furthermore, those experiencing the highest incidence of respiratory distress near the Salton Sea are primarily vulnerable populations that lack access to healthcare and are systemically barred from informing local policy, such as the Latinx and Indigenous Latin American (i.e., P’urhépecha) residents (Doede and DeGuzman, 2020; Rodriguez, 2021; Knoerr, 2022; Cheney et al., 2023). The rise in dust emissions coupled with the ongoing respiratory distress that overwhelmingly affects local marginalized communities highlights the connection between environmental health and public health, yet the role of aeolian dust microorganisms in this dynamic has yet to be explored.

While the major elemental composition of this dust has been investigated and no exceptional enhancement in toxic metals was found (Frie et al., 2017), there remains ongoing concern about dust composition, transport, and toxicity in the region. Notably, the microbial community of the dust has not been characterized, representing a major knowledge gap in terms of potential dust impacts. Here, we investigate the taxonomic and functional diversity of the aeolian dust microbiome sampled from the Salton Sea. We utilize 16S (V3-V4) rRNA amplicon sequencing to determine the microbial composition of the dust and shotgun metagenomics to assess the functional capacity and redundancy of the aeolian dust microbiome. We explore how dust sources and wind conditions in this region select for the assembly and survival strategies of the aeolian dust microbiome, and how its diversity may influence the health of nearby residents and ecosystems.

## Materials and methods

### Dust collection and processing

**FIGURE 1:**
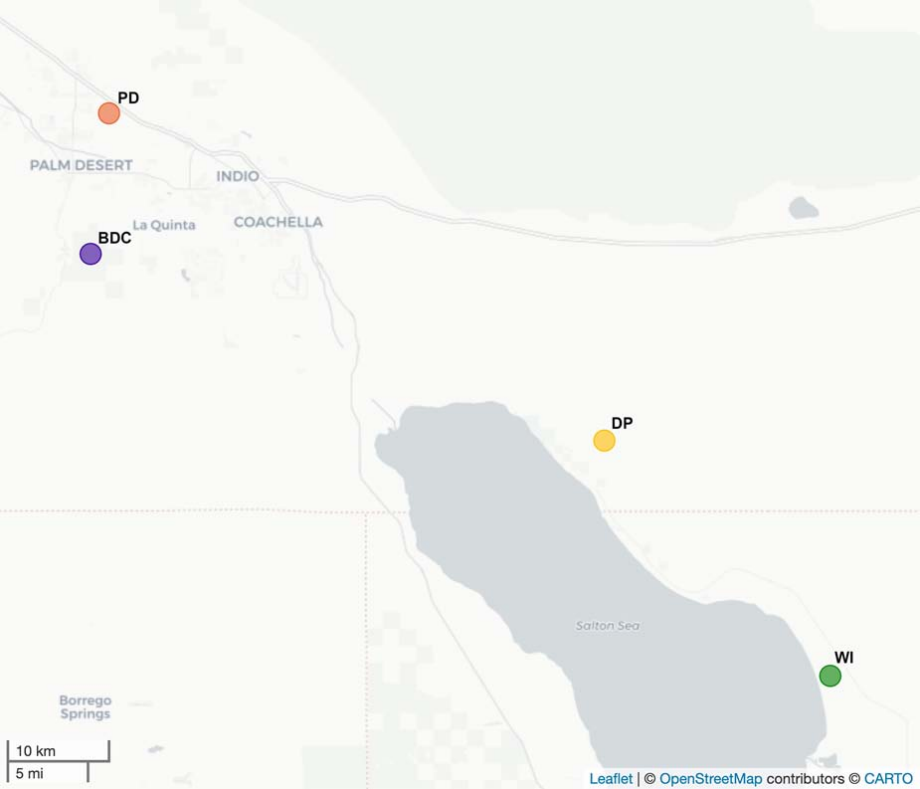
Map of the Salton Sea and the dust collection sites. This map displays the Salton Sea in Southern California, USA, and the four dust collection sites. “PD” represents the UC Riverside Palm Desert campus site (orange; 33.773808, -116.35286), “BDC” represents the Boyd Deep Canyon Desert Research Center site (purple; 33.6516667, -116.37264), “DP” represents the Dos Palmas Preserve site (yellow; 33.48859, -115.83517), and “WI” represents the Wister Recreation Area (green; 33.283861, -115.60008).

Passive dust collectors were used to capture aeolian dust from around the Salton Sea at four different sites during the Summer and Fall months in 2020 and 2021 (Figure 1 and Supplementary Table 1). Five replicate collectors were deployed at each of the following sites: the UC Riverside Palm Desert campus (PD; 33.773808, -116.35286), the Boyd Deep Canyon Desert Research Center (BDC; 33.6516667, -116.37264; Reserve DOI: (10.21973/N3V66D), the Dos Palmas Preserve (DP; 33.48859, -115.83517), and the Wister Recreation Area (WI; 33.283861, -115.60008). Dust collector deployment dates varied based on access to our collection sites, which was occasionally impeded by rain events. The sites were selected such that two sites, DP and WI, were located adjacent to the Salton Sea, and would be more likely to be impacted by dust and aerosols from the Sea, while the other two sites, PD and BDC, were located within the same region, but separated geographically from the Sea (Frie et al., 2019; Figure 1). The passive collectors consisted of Teflon-lined bundt pans (Nordic Ware, St. Louis Park, MN, United States) holding a Kevlar mesh (Industrial Netting Inc., Maple Grove, MN, United States) and filled with sterile glass marbles (12.7 mm diameter, Brooklyn, NY, United States; Aciego et al., 2017; Aarons et al., 2019). All materials used to assemble the passive dust collectors were first acid washed in 2M hydrochloric acid (i.e., HCl) followed by two subsequent rinses in ultrapure MilliQ water, and were always treated as such prior to re-deployment, then mounted on 8 foot poles in the field.

Upon retrieval from the field, passive dust collectors were placed in Whirlpak bags and stored in 4°C refrigerator for up to 24 h before they were processed in the lab. Each passive collector was rinsed with 1 L of sterile 18.2 Milli-Q water and used to create a dust suspension that was poured into1 L acid-washed Nalgene bottles (low density polyethylene), passing through a 2 mm buchner filter, which removed insects and/or large particulates. Dust suspensions were immediately subjected to vacuum filtration through an acid-washed, sterilized glass funnel using sterile 0.2 μm filters (47-mm diameter; Pall Supor 200 Sterile Grid filters, Pall Corporation, Port Washington, NY, United States) into an acid-washed collecting flask, as described in Maltz et al. (2022). A sterile dust collector was rinsed with 1 L 18.2 Milli-Q water and subjected to vacuum filtration using the same materials as our passive dust collectors. This sterile collector served as an experimental control, and their vacuum filtration filters were used in addition to unused filters to serve as controls for DNA extraction. For vacuum filtration of the dust suspensions and the control collector, 4–5 filters were used per liter of dust suspension. Filters were then stored frozen in sterile Whirlpak bags at –20°C until they could be used for DNA extraction.

### DNA extraction and library preparation

DNA extraction from the 0.2 μm filters were performed in duplicate with the Qiagen DNeasy PowerWater kit (Qiagen, Germantown, MD, United States). Two negative controls (i.e., two sterile 0.2 μm filters) were included during the DNA extraction process to confirm that the extracts were not contaminated. Raw DNA extracts along with negative DNA extraction controls were quantified with a NanoDrop 2000 (Thermo Fisher Scientific, Wilmington, DE, United States). The sterile dust collector filters did not contain DNA and thus were excluded from our pooled sequencing library. Half of the duplicate extracts were selected for purification based on their higher, raw DNA concentrations, and these extracts were purified via a bead clean-up using AMPure XP Beads and subsequently quantified with a NanoDrop 2000. Raw and purified DNA extracts were stored at -20 °C. Purified DNA extracts to be submitted for amplicon sequencing were then amplified via through a 2-step PCR using dual indices and sequencing adapters as per the Nextera XT Index kit (Illumina, San Diego, CA, United States). Specifically, Nextera-adapted Klindworth primers were used to target the 16S rRNA V3-V4 region (S-D-Bact-0341-b-S-17 and S-D-Bact-0785-a-A-21; Klindworth et al., 2013). Using the Nextera XT Index kit, each amplification reaction contained the following: 2.5 μL of DNA template, 5 μL each of the 1 μM forward and reverse index primers, and 12.5 μL of PCR KAPA HiFi HotStart Ready Mix to create a 25 μL reaction. Positive and negative controls were included in each step of the 2-step PCR, as well as in the sequenced libraries.

### DNA sequencing

The amplified DNA extracts for 28 samples and the two negative controls were pooled and sequenced via the Illumina, Inc. MiSeq platform (2 × 300 bp; Illumina 2017) by the UC Riverside Genomics Core. Twenty-four raw, non-amplified DNA extracts were sent on dry ice to the SeqCenter for shotgun metagenome sequencing. The SeqCenter prepared these libraries using the Illumina DNA Prep kit and IDT 10bp UDI indices and sequenced the libraries on an Illumina NovaSeq 6000 (2 × 151 bp). Adapters were trimmed by the SeqCenter using the bcl-convert v4.0.3. DNA extracts from samples that were collected in October 2020 were not submitted for shotgun metagenome sequencing due to extremely low DNA concentrations across samples and were thus used only for 16S rRNA amplicon sequencing.

### Bioinformatics—amplicon sequence data

Amplicon sequences were demultiplexed by the UC Riverside Genomics Core, and the FASTQ sequences were assessed for sequencing quality via FastQC (Andrews, 2010). In addition to FastQC, the eestats2 program (Edgar and Flyvbjerg, 2015) was used to determine the percentage of reads of specific lengths that will pass through the expected error threshold for a specific sample. The results supplied by FastQC and eestats2 were used to determine where the reads should be trimmed across the samples. Before trimming, there was a total of 11,646,700 reads (including forward and reverse reads) with a length of 301 base pairs across all 28 samples. The reads were then trimmed and filtered with BBDuk, a k-mer-based trimming and decontamination program from the BBTools suite created by the Joint Genome Institute (Bushnell, 2014), resulting in a total of 11,616,898 trimmed reads, ranging from 250 to 271 base pairs in length, across the samples. After trimming, the Divisive Amplicon Denoising Algorithm 2 (DADA2) pipeline (Callahan et al., 2016) was used via the RStudio environment (version 2023.03.0+386) to assign reads to amplicon sequence variants (ASVs). ASVs identified by the “decontam” package for R (Davis et al., 2018), as well as ASVs identified in the sequencing controls (which included our 2-step PCR positive and negative controls), were removed from the ASV count data. Decontam identified 2,156 ASVs which contained 941,426 reads, and our sequencing controls contained 62,506 ASVs with 478,665 reads attributed to these ASVs. Singletons and ASVs that were assigned to “Chloroplast” or “Mitochondria” taxonomic classifications were also removed from the ASV count data set. After using decontam and removing ASVs found in our sequencing controls, as well as removing eukaryotic reads, our samples contained 54,118 ASVs and a total of 1,332,482 reads. Before statistical analyses took place, the counts for each ASV were divided by the number of deployment days for that specific sample; this was performed to account for the variation in deployment duration across samples. The scaled counts were then multiplied by 100 and rounded to ensure that scaling by deployment did not skew the statistical analyses.

### Bioinformatics—metagenome sequence data

The sequence quality of the shotgun metagenomic data was assessed using FastQC (Andrews, 2010) and adapter and primer sequences were trimmed using BBDuk (Bushnell, 2014). After trimming, BBNorm (BBTools suite, Bushnell, 2014) was used to normalize the depth of read coverage in each metagenome. This normalization step ensures that there is an equal distribution of reads across all the sequenced regions, which is a necessary consideration with shotgun metagenomes due to their unequal sequence coverage (Bushnell, 2014). The normalized reads were then merged using BBMerge. The normalized reads were also error-corrected with SPades (Bankevich et al., 2012), and both the merged and non-merged, normalized reads were subsequently used for contig co-assembly with MEGAHIT (Li et al., 2015). Co-assembling contigs ensures that predicted genes identified by the functional annotation process are consistent across the metagenomes. The merged reads were also used to assist in the co-assembly of the contigs. The quality of the co-assembled contigs was determined using MetaQuast (Mikheenko et al., 2016). Trimmed, non-normalized metagenomic reads were then aligned to the co-assembled contigs using BWA-MEM (Li and Durbin, 2009; Li, 2013). After read mapping, contigs and scaffolds were binned into genomes (i.e., metagenome-assembled genomes; MAGs) with metaBAT (Kang et al., 2019), using the read mapping results from BWA-MEM to guide the binning. The quality and completeness of the MAGs were determined using CheckM (Parks et al., 2015). A custom bash script was then used to read the output from CheckM and parse out bins based upon their completeness and contamination, identifying high-quality bins (i.e., > 80% complete, < 5% contamination) for downstream analyses (Chen et al., 2020; Supplementary Table 2). Gene prediction was performed on the contigs and high-quality MAGs respectively using Prodigal (Hyatt et al., 2010). KOFamScan was then used to assign functions and KEGG orthologies (i.e., KO identifiers) to the predicted genes (Aramaki et al., 2020) in the contigs and high-quality MAGs. Gene assignments from KOFamScan were filtered so that each gene was assigned to a single KO identifier based on the lowest e-value for that gene assignment using the “bit-filter-KOFamScan-results” script from the “bit” package (Lee, 2022). Genes assigned the same KO ID are functional orthologs of one another, and thus code for the same function across organisms. High-quality MAGs were also taxonomically annotated using GTDB-tk (Chaumeil et al., 2020).

The number of metagenome reads that mapped to each gene in both the contigs and the high-quality MAGs was determined using featureCounts (Liao et al., 2014), which compares the alignment of the reads against the assembly (i.e., the co-assembled contigs or the MAGs) by BWA-MEM and the predicted genes found by Prodigal. The number of reads mapped to each gene calculated by featureCounts was merged with the functional annotations from KOFamScan via a custom bash script. The featureCounts results were then used to calculate depth of coverage for each gene in R by dividing the number of reads mapped to a gene from a sample by the gene’s length. This was done because different genes may be assigned the same KO identifier, and thus their gene length must be taken into consideration. These gene coverages were then divided by the number of days deployed for each sample to account for the variation in dust collector deployment. After scaling the gene coverages by deployment, these coverages were multiplied by 100 to ensure that the small gene coverages did not skew or interfere with the median of ratios normalization calculation (see below). Because multiple genes were assigned the same KO identifier(s), the scaled coverages for each gene assigned to the same KO were summed together to determine the total summed coverage per KO assignment within the contigs and high-quality MAGs respectively. The summed, scaled coverages per KO, per sample were subsequently normalized using the median of ratios normalization with the DESeq2 package in R to account for differences in sequencing depth across libraries (Love et al., 2014; Pereira et al., 2018; Xia, 2023). The normalized, summed coverages for the KOs will be hereafter referred to as normalized coverage throughout this manuscript.

The median of ratios normalization process calculates the geometric mean by each gene across the samples to create a reference that each raw gene count is divided by, generating a size factor for each sample. Each raw gene count (or coverage in our case) is then divided by this size factor to normalize the data. Because we were working with gene coverages that have been scaled by deployment days rather than raw counts, we decided to scale up the gene coverages by 100. Without this step, the normalization process is less effective due to the size factor value being equal to or larger than the coverage of most of the KOs in the metagenomes.

### Surface type frequencies and wind conditions

Estimates of likely source surfaces associated with PM during each collection period were calculated using hourly back-trajectories from the Hybrid Single-Particle Lagrangian Integrated Trajectory model (i.e., HYSPLIT), in combination with observed hourly PM data from nearby EPA surface stations (Environmental Protection Agency, 2024) and the gridded National Land Cover Database (NLCD) data product (Stein et al., 2015). Following Miao et al., 2024, for each hour of the collection period, we ran three HYSPLIT back trajectory simulations starting from three different heights within the typical daytime atmospheric boundary layer (100, 500, and 1,000 m). Each of those individual simulations then tracked air masses backwards through the 8 h immediately preceding its arrival at the collection site, generating a large footprint of potential surfaces contributing to collected particulate matter at that time. The resulting back trajectory points near the surface were then categorized based on a simplified set of NLCD surface types, including categories for barren land (i.e., Barren Land STF), crops (i.e., Crop Land STF), developed (i.e., urban; Developed STF), forest (i.e., Forest STF), herbaceous (i.e., Herbaceous STF), ocean (i.e., Ocean Water STF), and shrubs (i.e., Shrubs STF). The Salton Sea itself was also included as a distinct surface type (i.e., Salton Sea STF), representing possible emissions from the surrounding dried lakebed, as well as lake spray aerosol emitted directly from the water surface. Lastly, the “Others STF” category consists of land use types within NLCD that have extremely low frequencies for this region, including: “Hay/Pasture,” “Emergent Herbaceous Wetlands,” “Woody Wetlands,” and “Perennial Snow/Ice.” The frequencies of these surface categories were then weighted to account for factors influencing emissions and suspension, including wind speed, surface emissivity, and gravitational settling, and the resulting summed surface types were then applied to observed PM at each EPA surface station (Parajuli and Zender, 2017; Supplementary Table 3). This final data product, including a percent contribution from each surface type for every collection site and period, represents an estimate of likely source surfaces for collected PM, based on atmospheric dynamics and surface properties.

To retrieve publicly available weather data for the areas around our dust collector sites, weather stations were selected based on their proximity to the dust collector sites and the climate data available. Wind climate data from MesoWest/Synoptic network of weather stations was retrieved using the “mw” function from the “mesowest” package in R (Fick, 2018). Wind speed, wind direction, air temp, and relative humidity data was collected from the following weather stations: CI200 (nearest to our PD site), UCDE (nearest to our BDC) site, DPMC1 (nearest to our DP site), and CQ125 (nearest to our WI site). Accumulated precipitation data for a 24-h period was collected from the following weather stations: C2285 (nearest to our PD site), COOPDEEC1 (nearest to our BDC site), COOPMCAC1 (nearest to our DP site), and D3583 (nearest to our WI site). Each variable was measured every hour between our dates of interest. After these data were retrieved for our dates of interest, all variables excluding wind direction were averaged by our dates and times of interest, with each date corresponding to our dust collector deployment dates (with the deployment starting at 12:00 pm and collection ending at 5:00 pm; Supplementary Table 4). Wind direction was included by first converting reported wind angles and magnitudes into their vector components, including the meridional (north-south, v) and zonal components of the wind vector (east-west, u) contributions. These decomposed wind vector components were then averaged along with the other meteorological variables included here. Before surface type frequencies and climate data were used for statistical analyses, they were centered (i.e., each value was subtracted by its column mean) and scaled (i.e., dividing the centered values by their standard deviation) using the “scale” function from the “base” R package.

### Statistical analyses

The 16S rRNA amplicon data, the annotated contigs and MAGs, the surface type frequencies, and the wind conditions (i.e., average 24-h accumulated precipitation, average wind speed, average relative humidity, average wind direction) were analyzed in the RStudio environment using R software version 4.5.1. Correlations between the climate variables were compared using the “cor.test” function from the “stats” package and visualized using the “corrplot.mixed” function from the “corrplot” package (Wei and Simko, 2021; R Core Team, 2024). To determine if dust collector sites significantly varied by their climate conditions, the scaled surface type frequencies and wind condition data were log transformed, and the homogeneity of variance across sites were compared respectively using the “betadisper” function from the “vegan” package (Oksanen et al., 2020). Permutational multivariate analyses of variance (PERMANOVA) were performed with the “adonis2” function from the “vegan” package. All *p*-values for the PERMANOVAs were adjusted using the Bonferroni correction via the “p.adjust” function from the “stats” package. Additionally, specific site differences were explored using the “pairwise.adonis” function with 999 permutations from the “pairwiseAdonis” package (Martinez Arbizu, 2017).

Scaled, decontaminated 16S V3-V4 rRNA amplicon counts were rarefied to a sequencing depth of 3,200 via rarefaction using the “rrarefy” function from the “vegan” package. The sequencing depth used for rarefaction was the minimum number of total ASV counts observed across the samples, which was identified using the “min” and “rowSums” functions from the “base” package in R. Two samples with sequencing depths lower than 500 reads (i.e., PD.D.10.8.20 with a read depth of 227, WI.D.10.10.20 with a read depth of 162) were excluded from alpha diversity analyses. Shannon-Weiner diversity and species richness (i.e., alpha diversity) of the rarefied 16S V3-V4 rRNA amplicon count data (i.e., microbial composition data) were calculated using repeated rarefaction and averaging. To do this, the ASV counts were rarefied, then the Shannon-Weiner diversity or species richness was calculated using the “diversity” and “specnumber” functions from the “vegan” package, and this result was saved. This process was then repeated 100 times. Then the average of the Shannon-Weiner diversity and species richness measurements were calculated for each sample. Shannon-Weiner diversity and species richness were checked for normality via Shapiro-Wilks tests using the “shapiro.test” function from the “stats” package. The Shapiro-Wilks test determined that Shannon-Weiner diversity (*P <* 0.001) and species richness (*P <* 0.001) were not normally distributed, and thus, analyzing alpha diversity would require non-parametric statistical tests for this work.

The compositional relative abundance across samples was calculated using the scaled, decontaminated 16S V3-V4 rRNA amplicon counts and the “decostand” function (with “method = ‘total‘”) argument from the “vegan” package. The core aeolian dust microbiome was determined using the “core_members” and “plot_core” functions from the “microbiome” package (Lahti and Shetty, 2019), with a low detection (i.e., relative abundance in a sample) of 0.1% and a high detection threshold of 3%. Only microbial genera that had a minimum detection of 0.1% in at least 50% of the samples were considered members of the core microbiome. The justification for this detection threshold is two-fold: because dust is an extremely low biomass substrate, and because we did not want to exclude potentially rarer, yet widely distributed taxa from this analysis.

A Wilcoxon test was used to compare the mean of Shannon-Weiner diversity and species richness between sites respectively using the “wilcox.test” function from the “stats” package, and *p*-values were adjusted using the Bonferroni correction via the “p.adjust” function from the “stats” package. Additionally, a Kruskal-Wallis test was used to compare variance of alpha diversity and species richness between sites using the “kruskal.test” function from the “stats” package. After the Kruskal-Wallis, a Dunn test was used via the “dunn_test” function from the “rstatix” package to determine which sites’ variances were significantly different from one another (Kassambara, 2021). To then compare the homogeneity of variances across sites, a Fligner-Killeen test using the “fligner.test” function from the “stats” package was performed.

Beta diversity of the microbial composition data was performed by first transforming the data via a center-log ratio (i.e., CLR) transformation using the “decostand” function (with “method = ‘clr”’) from the “vegan” package. This function adds a pseudocount of 1 to all function counts, including those functions that have a count of zero, before performing the transformation. Then a Euclidean distance matrix of the CLR-transformed 16S V3-V4 amplicon count data was created using the “dist” function from the “vegan” package to calculate Aitchison distance, which was used as input to create a Principal Coordinates Analysis (i.e., PCoA) with the “pcoa” function from the “vegan” package. To determine how these samples cluster together, K-means clustering was performed using the “eclust” function from the “factoextra” package (Kassambara and Mundt, 2020). The gap statistic was calculated using the “fviz_gap_stat” and “fviz_nbclust” functions from the “factoextra” package to determine the ideal number of clusters (i.e., value for k).

Homogeneity of variance in the microbial composition data across sites were compared using the “betadisper” function from the “vegan” package. This was done using the Aitchison distances as input. Permutational multivariate analyses of variance (PERMANOVA) were performed with the “adonis2” function from the “vegan” package (with 10,000 permutations) to determine if there were significant differences in microbial composition across sites and depths. All *p*-values for the PERMANOVAs were adjusted using the Bonferroni correction via the “p.adjust” function from the “stats” package. Significant differences in microbial composition between specific sites was explored using the “pairwise.adonis” function from the “pairwiseAdonis” package using 9,999 permutations.

A Detrended Correspondence Analysis (i.e., DCA) was performed using the “decorana” function from the “vegan” package to determine if there was an arch effect present within the microbial composition data across sites and within sites. Due to the length of the first DCA axes, Redundancy Analysis (i.e., RDA) was chosen to determine if and how the microbial composition data were constrained by the climate data. RDAs were calculated using the “rda” function from the “vegan” package in the forward direction. The variation explained by the RDAs was obtained using the “RsquareAdj” function from the “vegan” package, and the significance of the RDAs was determined using the “anova” function from the “stats” package. The variance inflation factors for each predictor variable (i.e., the climate data) in the RDAs were determined using the “vif.cca” function from the “vegan” package. To find the best fitting model, the “ordistep” and “ordiR2step” functions from the “vegan” package were used. The “ordistep” function builds the RDA stepwise to determine which variables lead to significant changes in variance, using permutation *p*-values to assess the inclusion of each variable in the model. The “ordiR2step” function builds the RDA stepwise based on which variables maximize the adjusted variation explained by each predictor variable considered (i.e., their adjusted R^2^) and were statistically significant. To determine the contributed variation for each variable in the RDAs, variance partitioning was performed using the “varpart” function from the “vegan” package.

Generalized linear models (i.e., GLMs) were used to determine which climate variables (i.e., wind data and surface type frequencies) were significant predictors of specific functions of interest (i.e., KOs) found in the contigs from the metagenomes. Functions of interest were first chosen based on their involvement in dust microbial survival according to previous literature. Of these functions, KOs were selected for these models based on their scaled coverage (i.e., after median of ratios normalization) across the metagenomes, specifically choosing KOs that exhibited higher mean scaled coverage across the metagenomes. A Shapiro-Wilks test was used to see if these KOs were normally distributed across the metagenomes. For functions that were normally distributed, a linear regression was run using the “lm” function from the “stats” package, where the y variable was the scaled coverage for a specific KO and the x variable was a specific climate variable. For functions that were not normally distributed, generalized linear models were run using the “glm” function from the “stats” package using the Poisson distribution or a negative binomial distribution, where the y variable was the scaled coverage for a specific KO and the x variable was a specific climate variable. To determine the appropriate distribution for the non-normal dependent variables, a likelihood ratio test was performed using the lrtest() function from the lmtest package (Zeileis and Hothorn, 2002) and the Aikaike’s “An Information Criterion” (i.e., AIC) was calculated using the AIC() function from the stats package. Models with higher log likelihoods and smaller AIC values were then chosen as the best fit model for a particular formula. Significant predictors were identified by first iterating these GLMs in a stepwise fashion to identify which climate variables could be significant predictors of the distribution of these KO scaled coverages using the “step” function from the “stats” package. The “step” function runs these GLMs repeatedly, removing insignificant variables with every iteration, to determine which model has the lowest AIC. Based on the output of the stepwise GLM, the final GLMs were adjusted to only include significant climate predictors. After the final model for each function of interest was constructed, a Quantile-Quantile (i.e., Q-Q) plot of the models’ residuals was generated with the qqnorm() and qqline() functions from the stats package to assess their distribution. Additionally, the McFadden’s Pseudo R^2^ value for the negative binomial GLMs, as well as the GLMs using the Poisson distribution, was calculated to determine the variation explained by these models.

## Results

### Surface type frequencies and wind conditions by site

A PERMANOVA of the surface type frequencies (*P* = 0.003) and wind conditions (*P <* 0.001) showed that these conditions significantly varied by site respectively, while the dispersion of these data did not significantly vary (surface type frequencies, *P* = 0.704; wind conditions, *P* = 0.963). This indicates that these climate conditions were significantly different by site, and this difference cannot be attributed to the dispersion of these data by site alone. Further investigation into the differences in STFs between specific sites found that BDC and DP (*P* = 0.006), BDC and WI (*P* = 0.006), PD and DP (*P* = 0.006), and PD and WI (*P* = 0.006) varied significantly in their STFs. BDC and PD (*P* = 1) and DP and WI (*P* = 0.939) did not significantly vary from each other based on their STFs. All the sites significantly differed from each other based on their respective wind conditions. BDC and DP (*P* = 0.005), BDC and PD (*P* = 0.006), BDC and WI (*P* = 0.022), DP and PD (*P* = 0.002), DP and WI (*P* = 0.004), and PD and WI (*P* = 0.018) also varied significantly by their wind conditions.

### Microbial composition

Of the 54,118 ASVs identified, 3,306 bacterial and archaea genera were classified in our 28 samples. The fraction of Archaea identified in the samples were extremely low, with Archaea only being found in 10 of the 28 samples. The bacterial phylum Proteobacteria was the most relatively abundant across all the samples, followed by Firmicutes, Bacteroidota, and Actinobacteriota (Supplementary Figure 1). The bacterial order Burkholderiales was a major order (i.e., had a relative abundance of at least 5%) across all 28 samples. Bacterial genera and species that had a relative abundance of at least 10% in at least one sample include the following: *Acinetobacter baumannii, Rhizobium* sp., *Azohydromonas* sp., *Bacillus funiculus, Brevundimonas* sp., *Burkholderia* sp., *Candidatus Soleaferrea* sp., *Desulfovibrio cuneatus, Dyadobacter* sp., *Hymenobacter* sp., *Massilia* sp., *Nibribacter* sp., *Paenibacillus alvei, Panibacillus borealis, Pseudomonas lutea, Pseudomonas* sp., *Ramibacter* sp., *Ramlibacter* sp., *Roboutsia ilealis, Rosenbergiella* sp., *Spirosoma rigui, Variovorax paradoxus*, and *Zymobacter palmae.*

**FIGURE 2:**
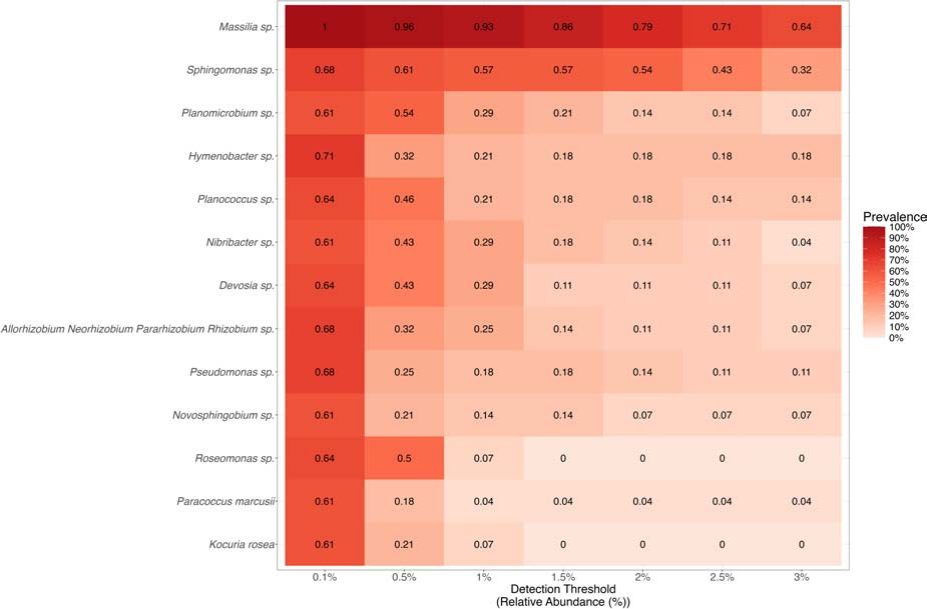
The core aeolian dust microbiome. This heatmap contains the 13 bacteria genera that compose the core aeolian dust microbiome in Salton Sea dust. The x-axis shows the detection threshold, which represents the relative abundance across all samples. The shade of red corresponds to the prevalence which describes how many samples contain each taxa. The prevalence value for a given taxa at a given detection threshold is also displayed in each cell of the heatmap.

A core aeolian dust microbiome was identified by examining which bacterial genera were detected across all samples (i.e., prevalence) at various relative abundances. These analyses revealed 13 bacterial genera with a relative abundances of at least 0.1% across more than 50% of the samples, which we call the core microbiome (Neu et al., 2021). From most to least abundant, these genera include: *Massilia, Sphingomonas, Planomicrobium, Hymenobacter, Planococcus, Nibribacter, Devosia*, *Rhizobium, Pseudomonas, Novosphingobium, Roseomonas*, *Kocuria*, and *Paracoccus* (Figure 2; Supplementary Figure 2). Only two taxa, *Kocuria rosea* and *Paracoccus marcusii*, were identified in this analysis at the species level. These 13 genera were found in 61% of the samples at a minimum of 0.1% the samples’ relative abundances. Major genera (i.e., genera with a relative abundance of at least 10% in one or more samples) that were identified in the core aeolian microbiome include *Massilia, Hymenobacter, Nibribacter, Rhizobium*, and *Pseudomonas*. *Massilia* was the most abundant genus overall; this genus represents one or more species that were member(s) of the core microbiome, with 64% of the samples containing *Massilia* taxa with a relative abundance of at least 3%. *Sphingomonas* was the only other genus to be observed at a relative abundance of at least 3 in 32% of the samples. The remaining 11 genera that were members of the core microbiome had relative abundances of at least 1 in 4–29% of the samples.

### Alpha diversity

Shannon-Weiner diversity (*P* = 0.68) and species richness (*P* = 0.5; Supplementary Table 5) did not significantly vary by site. This appears to be because of the within-sample variance exhibited in both alpha diversity and species richness across the sites (Supplementary Figures 3, 4). Alpha diversity and species richness did not significantly correlate with any of the surface type frequencies and wind condition data collected.

### Microbial beta diversity and its environmental drivers

**FIGURE 3:**
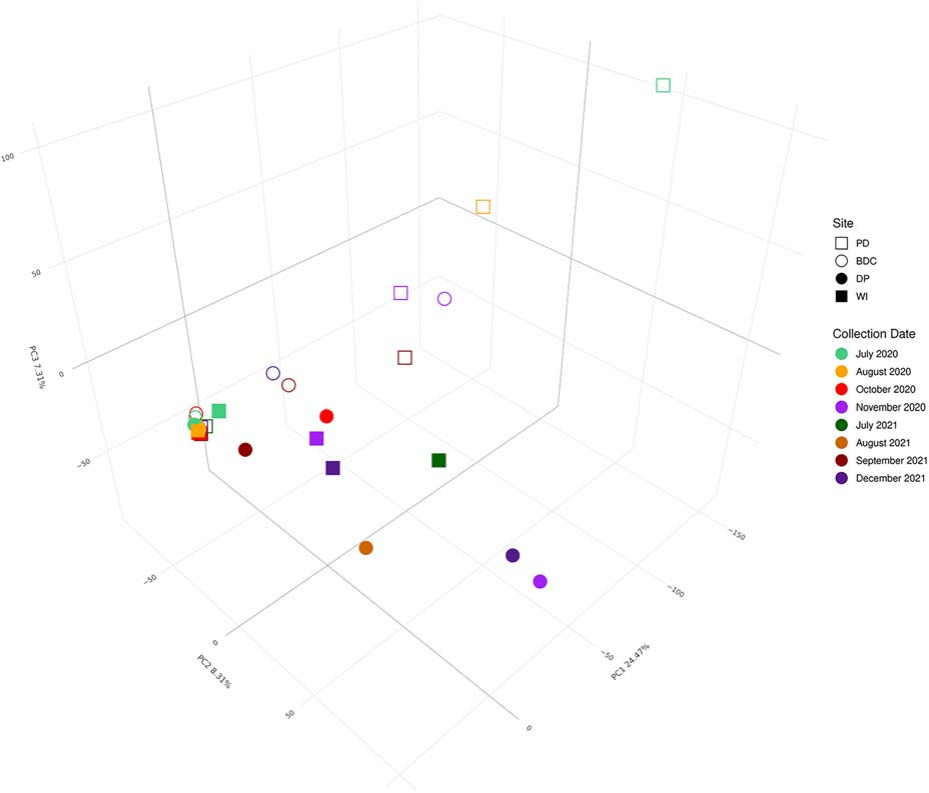
Principal coordinates analysis of microbial composition. This is a Principal Coordinates Analysis (i.e., PCoA) of microbial composition from the 16S V3-V4 rRNA amplicon sequence data. Each sample is represented by a point, where the shape corresponds to the collection site and the color is the collection date of that sample.

Microbial composition significantly varied between the four sites (*R*^2^ = 0.144, *P* = 0.046) and the dispersion across sites was homogenous (*P* = 0.342). A principal coordinates analysis (PCoA) (Figure 3) displays that while many samples cluster tightly together, samples from PD and BDC (i.e., sites closer to the Salton Sea) (Figure 1) lie along the first axis of variation (i.e., PC1), whereas DP and WI (i.e., sites closer to the Salton Sea) (Figure 1) lie on the second axis of variation (i.e., PC2). PC1 had a relative eigenvalue of 24.47%, indicating that this axis explained 24.47% of the total variation in microbial composition across the samples, which was attributed to samples from PD and BDC. PC2 had a relative eigenvalue of 8.31%, indicating that this axis explained 8.31% of the total variation in microbial composition, which was attributed to samples from DP and WI. K-means clustering (Supplementary Figure 5) revealed that the microbial composition forms three distinct clusters in these data: one cluster containing four PD samples and one BDC sample (i.e., PD.D.7.9.20, PD.D.8.14.20, BDC.D.11.6.20, PD.D.11.6.20, and PD.9.18.21) and another cluster containing three DP and two WI samples (DP.D.11.5.20, WI.D.7.29.21, DP.D.8.21.21, DP.D.12.8.21, and WI.D.12.8.21). While a PERMANOVA found that these four sites were significantly different in their microbial composition overall, a pairwise PERMANOVA found that the sites were not significantly different from one another (Supplementary Table 6). A PERMANOVA also revealed that beta diversity did not significantly differ between samples collected after rain events compared to samples collected during dry periods (*P* = 0.844).

**FIGURE 4:**
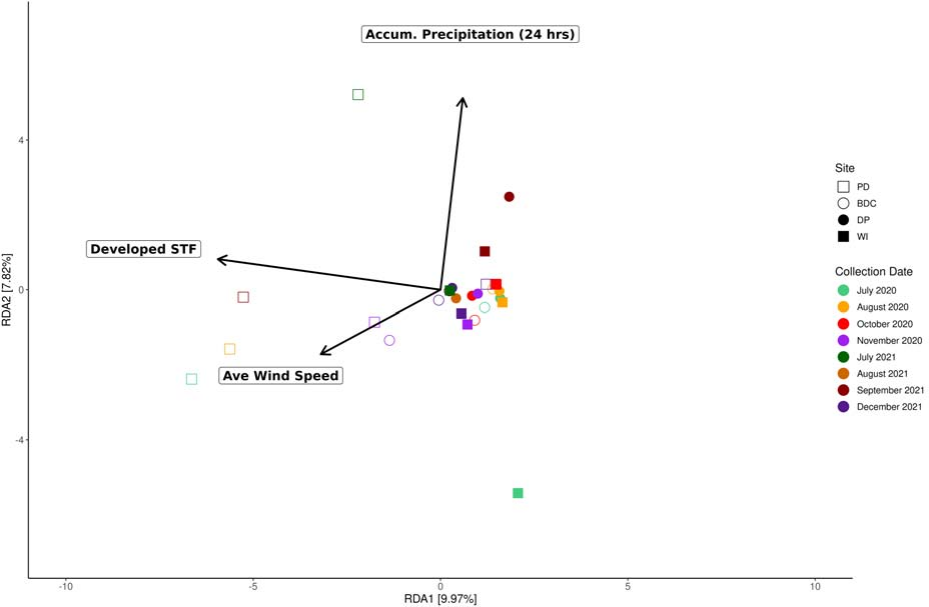
Environmental drivers of beta diversity. This redundancy analysis displays the significant environmental drivers of microbial composition across sites: the Developed STF (Adj *R*^2^ = 0.058, *P* = 0.001), average wind speed (Adj *R*^2^ = 0.02, *P* = 0.01), and the average 24-h accumulated precipitation (Adj *R*^2^ = 0.043, *P* = 0.033).

A redundancy analysis (i.e., RDA) of our samples found that the Developed STF (Adj *R*^2^ = 0.058, *P* = 0.001), the average wind speed (Adj *R*^2^ = 0.02, *P* = 0.01), and the average 24-h accumulated precipitation (Adj *R*^2^ = 0.043, *P* = 0.033) were significant drivers of microbial composition across sites (Adj *R*^2^ = 0.121, *P <* 0.001) (Figure 4 and Supplementary Table 7). Yet, these environmental variables were not necessarily significant drivers of microbial composition within each site. Microbial composition within the WI samples across time points was driven by average relative humidity (Adj *R*^2^ = 0.211, *P* = 0.02) and u, the zonal (east-west) component of local winds (Adj *R*^2^ = 0.167, *P* = 0.02). The microbial composition within DP samples were significantly driven by both average 24-h accumulated precipitation (Adj *R*^2^ = 0.179, *P* = 0.013) and the Barren Land STF (Adj R^2^ = 0.094, *P* = 0.034). Across the PD samples, average 24-h accumulated precipitation (Adj *R*^2^ = 0.29, *P* = 0.002) and average wind speed (Adj *R*^2^ = 0.22, *P* = 0.012) were significant environmental drivers of microbial composition. Lastly, average 24-hr accumulated precipitation (Adj *R*^2^ = 0.165, *P* = 0.045) and v, the meridional (north-south) component of local winds (Adj *R*^2^ = 0.101 *P* = 0.3), were significant environmental drivers of microbial composition in the BDC site across time points. While an ANOVA found the north-south wind component to be insignificant, the ordistep() function identified this variable as significant (*P* = 0.004).

### Metagenome sequence processing

2,130,808,324 reads were produced from the 24 shotgun metagenomes sequenced. After trimming with BBduk, 2,130,495,120 reads were used for contig assembly and genome binning. MEGAHIT yielded 4,947,761 co-assembled contigs. After mapping the trimmed reads to the co-assembled contigs, genome binning produced a total of 730 bins across the 24 metagenomes. A total of 1,059,642,227 reads from the 24 metagenomes were mapped to predicted genes identified in the co-assembled contigs. Of the 730 bins, 101 bins were considered high quality and used for taxonomic and functional annotation. The average genome completeness across the 101 bins was 93.81%, and the average contamination in the bins was 1.29%.

### Atmospheric survival functions, antibiotic resistance genes, and their depth of coverage in the co-assembled contigs

A total of 3,569,088 genes were identified by Prodigal, but not all genes were assigned corresponding KO IDs. After dropping genes that did not receive KO assignments, there was a total of 1,249,948 genes used to calculate the scaled gene coverages.

In total, 12,012 KO ID assignments were given to the 1,249,948 genes found across the contigs. After dropping KOs with low scaled, summed coverage (i.e., KOs with a scaled coverage of ≤3), 8,777 total KOs from the remaining identified genes in the contigs were normalized via median of ratios normalization and used for statistical analyses. Normalized, summed gene coverages of only KOs previously identified in aeolian dust microbiomes, and their pathway-associated genes, were used to determine if there is a core, aeolian dust microbiome at the functional level (DasSarma and DasSarma, 2018; Aalismail et al., 2019). Antibiotic resistance KOs considered here were previously identified in dust and other environmental samples, or belong to the same pathway of other antibiotic resistance genes found in environmental samples (Hu et al., 2018; Li et al., 2018; Marathe et al., 2018; Yang et al., 2018; Zhou et al., 2018; Maamar et al., 2020; Qin et al., 2022; Wang et al., 2022). These genes were chosen to represent each antibiotic drug group described on KEGG from their “Signature KOs for Antimicrobial Resistance” resource. For simplicity, the normalized, summed coverages per KO ID will be referred to as “normalized coverage” for the remainder of this manuscript.

**FIGURE 5:**
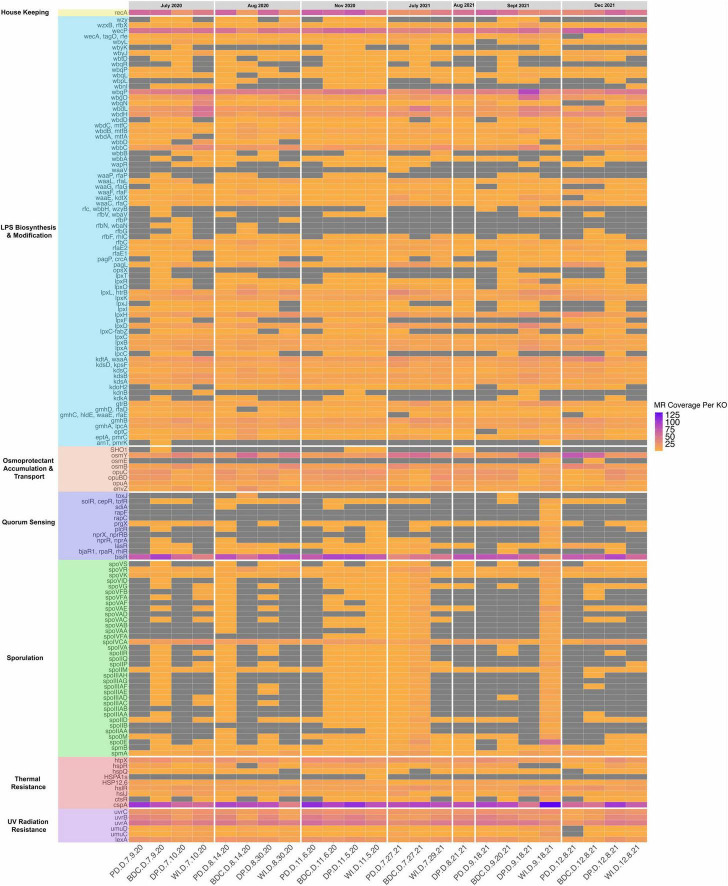
This heatmap shows the normalized coverage of KO IDs involved in surviving the atmospheric environment found in the metagenomes. The genes are grouped by functions in this order: housekeeping gene, lipopolysaccharide (LPS) biosynthesis/modification genes, osmoprotectant transport/accumulation, quorum sensing genes, sporulation genes, temperature shock response genes, and UV-damaged DNA repair genes. The x-axis contains metagenome sample IDs, and the y-axis contains KO IDs.

Genes assigned to KOs involved in endospore formation, UV-damaged DNA repair, the temperature shock response, osmoprotectant transport and accumulation, lipopolysaccharide (LPS) synthesis and modification, quorum sensing, and antibiotic resistance were found in all 24 metagenomes. These atmospheric survival genes and their normalized coverages can be found in Figure 5. As for genes involved endospore formation, four of the 38 sporulation genes (i.e., the spore maturation protein A—*spmA*; site-specific DNA recombinase—*spoIVCA*, stage V sporulation protein K—*spoVK*, and stage V sporulation protein R—*spoVR*) were found in all 24 metagenomes while the remaining sporulation genes included here were absent.

**FIGURE 6:**
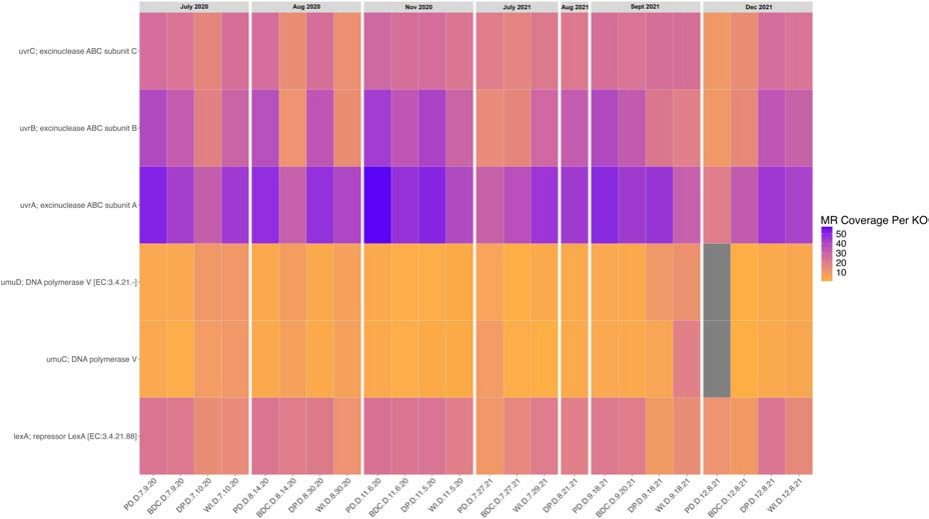
Heatmap of UV-radiation resistance in aeolian dust metagenomes. This heatmap shows the normalized coverage of KO IDs involved in repairing UV-damaged DNA across the metagenomes. Each column is a metagenome (i.e., one sample), and the metagenomes are organized by their collection dates from July 2020 to December 2021. The x-axis contains sample IDs, and the y-axis contains gene names for each KO.

UV-damaged DNA repair genes were also found in all 24 metagenomes, though their normalized coverages were not evenly distributed (Figures 5, 6). Excinuclease subunits ABC (i.e, *uvrA*, *uvrB*, *uvrC*) were at found at higher coverages than the repressor LexA (i.e., *lexA*) and DNA polymerase V (i.e., *umuC, umuD*) across the metagenomes. Due to the presence and distribution of these genes in all 24 metagenomes, a PERMANOVA was calculated to determine if these genes significantly varied in their normalized coverages by site and collection date. The PERMANOVA found that the normalized coverages of the UV-damaged DNA repair genes in the metagenomes did not significantly vary by site (*P* = 0.646) or collection date (*P* = 0.350).

Genes involved in regulating heat and cold shock responses were found in all 24 metagenomes. Heat shock proteins HtpX (i.e., *htpX*), 12.6 (i.e., *HSP12.6*), and ribosome-associated Hsp15 (i.e., *hsIR*) were found across the metagenomes. Additionally, the cold shock protein (i.e., *cspA*) was found in every metagenome at a higher the normalized coverage compared to the other temperature shock-related proteins examined in this work. Other temperature shock-related genes found in the metagenomes include heat shock protein HspR (i.e., *hspR*), heat shock protein HsQ (i.e., *hspQ*), and a transcriptional regulator of stress and heat shock response known as *ctsR*. Due to the distribution and prevalence of these genes across all 24 metagenomes, a PERMANOVA was calculated to clarify if these genes significantly varied in their normalized coverages by site and collection date. The PERMANOVA revealed that the normalized coverages of the temperature shock response genes did not significantly differ by site (*P* = 0.840) or by collection date (*P* = 0.637).

Osmoprotectant transport and accumulation genes were also shared across the 24 metagenomes. The hyperosmotically inducible periplasmic protein (i.e., *osmY*), osmotically inducible lipoprotein OsmB (i.e., *osmB*), osmoprotectant transport system substrate-binding protein (i.e., *opuC*), osmoprotectant transport system permease protein (i.e., *opuBD*), osmoprotectant transport system ATP-binding protein (i.e., *opuA*), and the osmolarity sensor histidine kinase of the OmpR family EnvZ (i.e., *envZ*) were found in all the metagenomes. Due to the distribution of most of the osmoprotectant transport/accumulation genes in the metagenomes, a PERMANOVA was calculated to determine if these genes were significantly different in their normalized coverages by site and collection date. The PERMANOVA revealed that the normalized coverages of the osmoprotectant transport/accumulation genes found did not significantly differ by site (*P* = 0.831) or by collection date (*P* = 0.296).

LPS synthesis and modification genes were widely distributed across the metagenomes. All genes that are involved in constructing or modifying various segments of LPS considered here were found in at least one of the 24 metagenomes. Most of the genes that contribute to building or modifying the core region and the lipid A portion of LPS were found in at least 12 of the 24 metagenomes. Genes involved in constructing or modifying the O-antigen repeat unit within the LPS were widely distributed across the metagenomes, with two genes exhibiting the highest normalized coverages of the LPS genes considered: the UDP-GalNac:undecaprenyl-phosphate GalNac-1-phsophate transferase (i.e., *wecP*) and the O55-antigen biosynthesis glycosyltransferase (i.e., *wbgP*).

Quorum sensing genes were also found in the metagenomes, though only one gene was shared across all 24 metagenomes: the LuxR family transcriptional regulator/ quorum-sensing system regulator BisR (i.e., *bisR*). Other genes in the LuxI/LuxR acyl-homoserine lactone (i.e., AHL) quorum sensing system identified in the metagenomes include the LuxR family transcriptional regulator/quorum-sensing system regulators BjaR1 (i.e., *bjaR1*, 12 of 24 metagenomes), LasR (i.e., *lasR*, 20 of 24 metagenomes), and SolR (i.e., *solR*, 16 of 24 metagenomes). The HTH-type transcriptional regulator/major conjugation operon repressor known as *prgX*, which is involved in the Rap/NprR/PlcR/PrgX (i.e., RNPP) pathway, was found in 20 of the 24 metagenomes.

**FIGURE 7:**
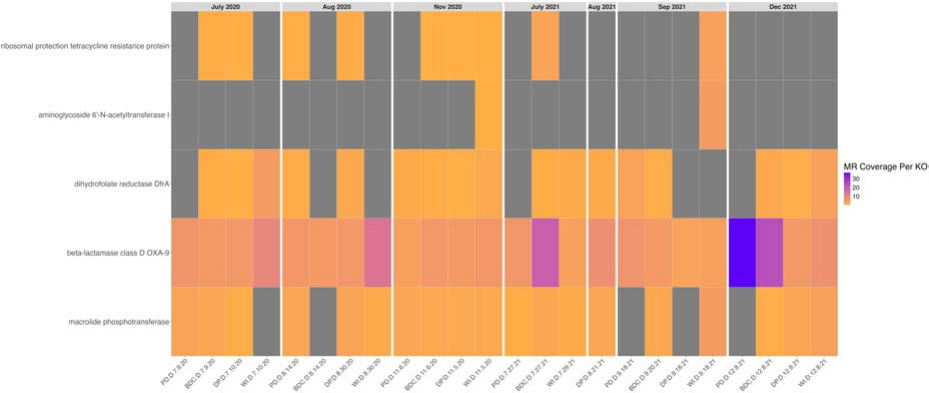
Heatmap of antibiotic resistance genes in aeolian dust metagenomes. This heatmap shows the normalized coverage of KO IDs assigned to antibiotic resistance genes across the metagenomes. Each column is a metagenome (i.e., one sample), and the metagenomes are organized by their collection dates from July 2020 to December 2021. The x-axis contains sample IDs, and the y-axis contains gene names for each KO.

Antibiotic resistance genes (i.e., ARGs) (Figure 7) were identified in all 24 metagenomes, specifically the following genes: ribosomal protection tetracycline resistance protein (in 9 metagenomes), macrolide phoshotransferase (in 19 metagenomes), dihydrofolate reductase DfrA (in 17 metagenomes), beta-lactamase class D OXA-9 (in 24 metagenomes), and aminoglycoside 6’-N-acetyltransfer I (in 2 metagenomes). Of the ARGs considered here, only beta-lactamase class D OXA-9 was found in every metagenome.

### Surface type frequencies, wind conditions, and weather as predictors of dust microbiome function

**FIGURE 8:**
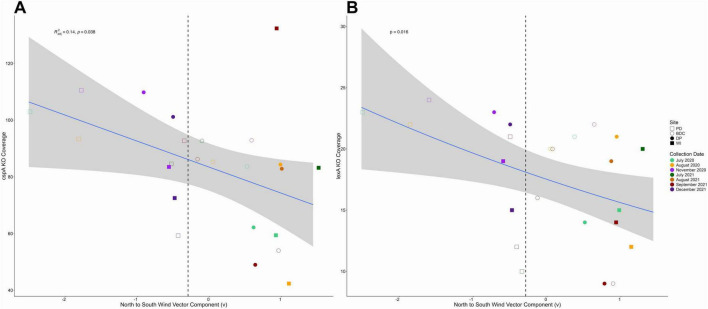
Meridional wind component (v) predicts *cspA* **(A)** and *lexA* **(B)** in dust metagenomes. This scatterplot shows how the meridional component (v) of local winds significantly predicts the normalized coverage of the cold shock protein *cspA* and the repressor *lexA*. Points to the left of the dashed line are predominantly samples that received northerly winds, and points to the right of the dashed line include samples that received southerly winds.

After identifying the core aeolian microbiome functions of interest in the metagenomes, certain KOs were selected for generalized linear models based on their normalized coverages compared to other KOs in their adaptation category (Supplementary Table 8). The KOs chosen had the highest mean normalized coverage within their respective categories across the metagenomes. *spoIVCA* and *spmA* represented sporulation genes, *lexA* (Figure 8), *uvrA* (Supplementary Figure 6), *uvrB* (Supplementary Figure 6), and *uvrC* represented the genes responsible for UV-damaged DNA repair, *cspA* (Figure 8) and *htpX* represented the temperature shock response genes, *osmY* and *opuC* represented genes involved in osmoprotectant transport and accumulation, *wecP* and *wbgP* represented genes involved in LPS modification and synthesis (of the O-antigen repeat unit), and *bisR* and *prgX* represented genes involved in quorum sensing pathways.

### Taxonomic annotation of the metagenome-assembled genomes

The 101 high-quality MAGs (Supplementary Table 9) were assigned to the Bacteria domain and to the following phyla: Proteobacteria (*n* = 44), Firmicutes (*n* = 27), Actinobacteriota (*n* = 19), Bacteroidota (*n* = 7), Spirochaetota (*n* = 3), and Desulfobacterota (*n* = 1). The Proteobacteria MAGs were assigned to the Gammaproteobacteria (*n* = 30) and Alphaproteobacteria (*n* = 14) classes. Firmicutes MAGs were assigned to the Bacilli (*n* = 25) and Clostridia (*n* = 2) classes. All 19 Actinobacteriota MAGs were assigned to the Actinomycetia class, and all 7 Bacteroidota MAGs were assigned to the Bacteroidia class. The 3 Spirochaeota MAGs were all assigned the same identity (i.e., Spirochaetia class, Borreliales order, and Borreliaceae family), but were not classified beyond the family level. Lastly, the MAG within the Desulfobacterota was assigned to the genus *Frigididesulfovibrio* (i.e., Desulfovibrionia class, Desulfovibrionales order, Desulfovibrionaceae family).

88 of the 101 MAGs were identified at the genus level, and 37 MAGs were identified at the species level (Supplementary Table 9). The 37 MAGs identified at the species level include the following taxa: *Corynebacterium sp012838715* (n = 12), *Salinicoccus roseus* (*n* = 11), *Acinetobacter baumannii* (*n* = 2), *Bartonella sp016102265* (*n* = 2), *Priestia megaterium* (*n* = 2), *Brevundimonas vesicularis* (*n* = 1), *Cereibacter changlensis* (*n* = 1),*Curtobacterium sp001705035* (*n* = 1), *Cutibacterium acnes* (*n* = 1), *Enterobacter kobei* (*n* = 1), *Exiguobacterium acetylicum* (*n* = 1), *Gilliamella apicola* (*n* = 1), and *Pseudarthrobacter phenanthrenivorans* (*n* = 1).

### Atmospheric survival functions, antibiotic resistance genes, and their depth of coverage in the metagenome-assembled genomes

As was done with the co-assembled contigs, genes assigned to KOs previously identified in aeolian dust microbiomes were also identified in the MAGs. Genes assigned to KOs involved in UV-damaged DNA repair, the temperature shock response, osmoprotectant transport and accumulation, lipopolysaccharide (LPS) synthesis and modification, and quorum sensing were found in 95 of the 101 MAGs (Supplementary Figure 7). Certain functions of interest were more widely distributed than others across the MAGs. 88 of the 95 MAGs contained the *cspA* gene; these bins were assigned to both Gram-negative and Gram-positive bacteria and were isolated from all four sites. In addition to *cspA*, other temperature shock genes shared across the MAGS included *hsIR* (i.e., a gene that codes for the ribosome-associated heat shock protein Hsp15) in 67 MAGs and *htpX* in 39 of the MAGs. As for genes involved in UV-damaged DNA repair, 87 of the 95 MAGs contained the *uvrC* gene, 85 of the 95 MAGs contained the *uvrB* gene, and 83 of the 95 MAGs contained the *uvrA* gene (Supplementary Figures 7, 8). Genes involved in osmoprotectant transport and accumulation were shared across the metagenomes as well, with 35 MAGs containing *opuC* and 28 MAGs containing *osmY*. 12 of the 95 MAGs contained at least 18 of the 35 sporulation genes examined here. LPS development genes included *wbgP* in 51 MAGs, *gtrB* (i.e., polyisoprenyl-phosphate glycosyltransferase; involved in O-antigen modification) in 43 MAGs, *wecP* in 41 MAGs, and *lpxD* (i.e., UDP-3-O-[3-hydroxymyristoyl] glucosamine N-acyltransferase; involved in Lipid A synthesis) in 41 MAGs. Though most of the quorum sensing genes and sporulation genes considered here were not widely distributed across the MAGs, *bisR* was found in 53 of the MAGs, *spoVR* (i.e., codes for the stage V sporulation protein R) was found in 25 MAGs, and *spmA* was found in 24 MAGs. Lastly, ARGs were not widely distributed in the MAGs and only appeared in 12 MAGs (Supplementary Figure 9). Of the ARGs identified, the most prevalent was the macrolide phosphotransferase gene, found in eight MAGs.

## Discussion

Here we investigate the microbial composition and functional diversity of the previously uncharacterized dust microbiome from the Salton Sea region. Despite significant differences in the dust sources and the wind conditions of our four sites (i.e., PD, BDC, DP, and WI) across several months in 2020 and 2021, we observed a core aeolian microbiome in both compositional and functional assembly. Additionally, the dust sources, wind conditions, and weather in this region contributed to the overall composition of the dust microbiome as well as specific adaptations required for aeolian microbial survival. Collectively, our results suggest that the influence of the local climate as well as the resilience of the microorganisms entrained in the dust work in tandem to structure the taxonomic and functional diversity of the core, aeolian dust microbiome.

### Dust sources and local wind conditions drive dust microbiome assembly

We observed that aeolian dust microbial composition from around the Salton Sea significantly varied between our sites: PD, BDC, DP, and WI. While Shannon-Weiner diversity and species richness did not significantly differ between sites (Supplementary Figures 3, 4), we did observe a difference in the distribution of specific bacterial genera between sites further from (i.e., PD, BDC) and nearer to (i.e., DP, WI) the Salton Sea (Supplementary Figure 5). These results suggest that samples from sites further from the Salton Sea (i.e., PD and BDC) and sites closer to the Salton Sea (i.e., DP and WI) may share many microorganisms, but their respective communities are heavily regulated by different taxa. We also found that dust sources, local wind conditions, and weather differentially structured the dust microbiomes within each site. These findings indicate that the chemical composition of the dust and the source of the dust work in tandem as stringent selective filters that differentially determine which microorganisms become entrained, persist, and disperse in aeolian dust around the Salton Sea and beyond. Previous research into the chemical composition and sources of dust collected around the Salton Sea has shown that dust composition and source vary depending on where the dust was collected. Frie et al. (2019) collected aeolian dust at our four sites (PD, BDC, DP, and WI) as well as a fifth site (Sonny Bono; SB) and discovered that evaporite-associated elements (i.e., Na, Ca, K, Mg, Sr) were most enriched in sites nearest to the Salton Sea (i.e., WI, DP) and decreased in concentration as the dust traveled from south to north (Frie et al., 2019). Conversely, elements associated with soil crusts (i.e., Ti, Fe, Co, Ba, Sn) exhibited the opposite trend, with the highest enrichment at PD and BDC, and decreasing as the dust moved south (Frie et al., 2019). Considering that nutrient availability and distance are important factors in guiding microbial dispersal and assembly, our results demonstrate that local wind conditions and sources select for the variation in taxonomic assembly of dust microbiomes in the Salton Sea region.

### Aeolian dust has a core microbiome based on composition

Despite the differences in aeolian dust microbial composition between our four sites, we observed a core aeolian dust microbiome composed of thirteen bacterial genera. Our results demonstrate that aeolian dust from around the Salton Sea shares a set of unique microorganisms that can withstand the harsh conditions of the atmosphere. Twelve of the thirteen bacterial genera identified in the Salton Sea core dust microbiome have been previously identified in dusts and aerosols from around the world. Of these thirteen taxa, *Massilia* dominated our samples and appeared to be the most abundant member of the core dust microbiome. This Gram-negative genus was previously identified in the core microbiome of airborne dust in Kuwait (Al Salameen et al., 2020), dust samples from the Eastern Mediterranean (Erkorkmaz et al., 2023), dust rains in Beirut, Lebanon and Granada, Spain (Itani and Smith, 2016; Navarro et al., 2023), and air samples collected in the Suwon region of South Korea (Weon et al., 2008, p. 20). *Massilia* is of particular interest due to its presence in all our samples and its known resiliency against both hot and cold temperatures as well as UV-radiation stress. *Massilia* species are known to be pyrophilous, heat resistant, and desiccation resistant, dominating forest soils immediately after a fire (Pulido-Chavez et al., 2023), and have been isolated from desert soil crusts, photovoltaic cells (Moura et al., 2021), and arctic microbial mats (Shaffer et al., 2023). Our results were consistent with these studies’ findings, and comprehensively, these results speak not only to *Massilia*’s ubiquity in atmospheric samples globally, but also to this genus’ ability to readily adapt to its harsh, dynamic conditions. Given the presence and abundance of *Massilia* species in every sample, regardless of collection period, coupled with its weather-resistant adaptations, it is evident that *Massilia* is the quintessential microorganism of the aeolian dust microbiome.

In addition to *Massilia*, other members of the core dust microbiome have been isolated in a variety of environmental dust and soil surface samples. Air samples collected at Peking University in Beijing, China contained nine of our 13 core dust bacteria: *Sphingomonas, Hymenobacter, Planomicrobium, Pseudomonas, Novosphingobium, Roseomonas, Paracoccus, Kocuria*, and *Massilia* (Zhang et al., 2019b). Bacterial cultures isolated from dust collected in Ilam city, Iran, contained *Pseudomonas, Planococcus, Paracoccus*, and *Rhizobium* colonies (Amarloei et al., 2020). Additionally, a global study of settled dust (i.e., including samples from 33 countries and six continents) found that not only did *Sphingomonas* and *Hymenobacter* dominate their dust samples, but they also identified the presence of *Devosia*, *Paracoccus*, and *Pseudomonas* in their dust microbiomes (Chen et al., 2021). Lastly, while *Nibribacter* has not been explicitly identified in dust, this genus was abundant in emissive surface sand from the Kyzyl-Kum desert in Uzbekistan along with other known dust-inhabiting microorganisms such as *Roseomonas, Hymenobacter, Novosphinogobium, Planococcus, Planomicrobium, Sphingomonas*, and *Massilia* (Osman et al., 2023). In summary, our results offer further support that the core bacteria we identified could persist in the harsh and volatile conditions of aeolian dust.

### Aeolian dust has a core microbiome based on function

Functional annotation of the aeolian dust metagenomes sampled from the Salton Sea revealed that the aeolian dust microbiome in this region is equipped to survive atmospheric conditions. Genes that code for proteins involved in endospore formation, UV-damaged DNA repair, the temperature shock response, osmoprotectant transport and accumulation, lipopolysaccharide (LPS) synthesis and modification, and quorum sensing were widely distributed across genes our metagenomes as well as MAGs. The presence and distribution of these traits throughout the metagenomes from all four sites indicates that the aeolian dust microbiome contains the set of conserved features required to withstand entrainment and colonization of the atmospheric ecosystem. Previous studies into the functional diversity of atmospheric microbiomes have found similar traits that are necessary for windblown survival and dispersal. Metagenomes from air samples collected over the Red Sea contained genes that code for proteins involved in UV-radiation resistance (i.e., *uvrA*), quorum sensing and biofilm formation genes (i.e., *vpsT*), heat shock resistance (i.e., *HSP70, HSP90*), and sporulation (i.e., *spoVK*, *spo*IVFB; Aalismail et al., 2019). Bacteria sampled from clouds (Joly et al., 2015), rainwater (Ling et al., 2021) and air (Daussin et al., 2023) have been shown to resist a variety of stressors associated with wind and the atmosphere including UV-radiation, osmotic stress, and the freeze-thaw cycle. Thus, we can conclude that the dust microbiome in the Salton Sea region has the functional capacity and redundancy to withstand the atmospheric stressors associated with aeolian entrainment, colonization, and dispersal.

### Wind direction and seasonality select for microbial functional adaptations

We find that while the aeolian dust microbiome has a core set of required adaptations for survival, a single abiotic factor cannot explain the prevalence and distribution of these functions. Our results highlight the complexity of the aeolian environment and its multiple selective pressures that synergistically structure the functional diversity of dust microbiomes. The average meridional (north-south) components (v) and zonal (east-west) components (u) of local winds were significant or near significant predictors for several essential dust microbial traits. Notably, the meridional (north-south) component (v) was the sole significant predictor for functions involved in UV-radiation resistance (i.e., *lexA*) and thermal resistance (i.e., *cspA*). The meridional (north-south) component (v) was also a significant predictor in conjunction with other meteorological variables of several traits including UV-radiation resistance genes *uvrA, uvrB*, and *uvrC*, sporulation gene *spmA*, and osmotic stress resistant gene *osmY* (Supplementary Table 8). When plotting the normalized coverage of these functions against the meridional (north-south) component, a seasonal trend emerged: the UV-radiation resistance genes (specifically *lexA*, *uvrA*, and *uvrB*) and *cspA* appeared to be more prevalent with northerly winds that dominated the winter months compared to the lower coverage with southerly winds that occurred in the summer and fall months. These seasonal weather patterns have been well described, with northerly winds at the Salton Sea occurring in the winter and southerly winds occurring in summer and fall (Frie et al., 2017, 2019). These trends observed in the conserved adaptations across sites highlight the functional plasticity, redundancy, and resiliency of the aeolian dust microbiome throughout changing weather conditions over time.

We postulate that in the fall and winter months, when dust flux, wind speeds, and air temperatures were low, the generalist aeolian dust microbiome thrived unhindered by the local weather. As wind temperatures and speeds rose in spring and summer, the wind direction shifted and the contribution of Salton Sea playa dust and sea spray into the atmosphere increased. Together, these conditions disturbed the generalist aeolian microbiome and potentially introduced Salton Seawater microorganisms into the atmosphere. Aeolian microbiomes have been shown to exhibit compositional and functional differences during days with and without dust events, and these microbial communities significantly differed from one another based on their respective dust sources (Gat et al., 2017; Maltz et al., 2022; Erkorkmaz et al., 2023). Furthermore, these microbial communities have demonstrated compositional recovery after a dust storm. For instance, Mazar et al. (2016) found that dust microbial composition significantly varied between dust event collections and clear days despite collecting these samples from a single location (Mazar et al., 2016). Thus, it is plausible that the ambient dust microbiome we sampled may have been disturbed by incoming Salton Sea dust and sea spray during the summer months and was able to recover post disturbance in the fall and winter. We did identify microbial genera that were shared between Salton Sea dust and seawater samples (Freund et al., 2025), which suggests that the lake water and dust within this ecosystem interact with one another. Furthermore, we observed the seasonal recovery of necessary survival traits (i.e., UV-radiation resistance genes, *cspA*) in the aeolian microbiome across 2020 and 2021, suggesting that the aeolian microbiome is readily adaptable and ecologically resilient.

Despite dust microbial adaptations exhibiting a seasonal shift in their distributions, the relative abundance of core dust microbial genera did not follow a seasonal trend. This functional convergence and taxonomic divergence could be due to horizontal gene transfer events (i.e., HGT) within the aeolian dust microbiome, contributing to the acquisition of atmosphere-specific adaptations in microorganisms beyond those in the core dust microbiome. This observation led us to consider if evidence of HGT could be identified in our metagenomes. HGT events have been traced by using specific marker genes that are known to be shared via this process such as the Class I integron-integrase gene (i.e., *intI1*) and antibiotic resistance genes (i.e., ARGs), many of which have been observed in dust microbial communities (Gat et al., 2017; Li et al., 2018; Zhang et al., 2019a; Maamar et al., 2020). We did identify the presence of several ARGs in all 24 aeolian dust metagenomes and in 12 MAGs, including ARGs that target different antibiotics and revealing the capacity for multi-drug resistance in the Salton Sea dust microbiome. Of note is beta-lactamase class D OXA-9, which was found in every metagenome; beta-lactamases have been found to be associated with plasmid mobilization and HGT, and they have been found in environmental microbiomes, including atmospheric microbiomes (Hu et al., 2018; Li et al., 2018; Marathe et al., 2018, p. 201; Yang et al., 2018; Zhou et al., 2018; Maamar et al., 2020). The identification of multi-drug ARGs in the aeolian dust microbiome may indicate that HGT is a process that confers resistance or resilience to both competing microorganisms and fluctuating weather conditions, enhancing their viability and dispersal ability. Furthermore, the presence of multi-drug ARGs in the dust microbiome is alarming because residents in the area exposed to this dust subsequently experience respiratory distress and other health complications (Farzan et al., 2019; Jones and Fleck, 2020; Miao et al., 2024; Maltz et al., 2025). Once dust microorganisms enters the host’s airway upon inhalation, they can interact and potentially exchange genes with host’s microbiomes, including ARGs, via HGT (Aogáin et al., 2020; Bai et al., 2024). The transfer of these ARGs and hitchhiker genes from the dust microbiome to host microbiomes could reduce the efficacy of antibiotics used to treat infections in dust-exposed patients near the Salton Sea and select for the proliferation of host pathobionts, thus increasing the susceptibility of exposed individuals to a wide variety of health issues including respiratory illnesses and lung dysbiosis. Research has shown that individuals with respiratory illnesses such as chronic obstructive pulmonary disease (i.e., COPD) and bronchiectasis exhibited respiratory dysbiosis and an increased abundance of certain ARGs compared to healthy individuals (Aogáin et al., 2020). The distribution and dispersal of ARGs because of HGT has been well studied due to its impact on the pathogenicity of certain microorganisms, yet the sharing of concomitant, niche-specific adaptations via HGT within and between environmental and host microbiomes requires further study to assess their effects (Fuchsman et al., 2017, p. 201). Future work exploring HGT events within the Salton Sea dust microbiome is required to clarify the selective mechanisms behind the functional convergence and taxonomic divergence we observed in this microbial community, and how these selective pressures influence the potential pathogenicity of the aeolian microbiome.

### Study limitations

While this study is the first to explore the microbial diversity of dust from the Salton Sea, this work does have its limitations. One limitation of this study is that dust collectors were not deployed for the same amount of time, due to weather events that made retrieving our passive dust collectors impossible. To account for this uncontrollable factor, we decided to divide bacterial ASV counts in each sample by the number of days that a particular sample (i.e., single dust collector) was deployed for, which is described in our methods section. Several studies have shown that increases in dust deposition correspond to increases in bacterial biomass and bacterial metabolic activity (Tsiola et al., 2017; Bigelow et al., 2020; Zhao et al., 2025). Thus, while we could not quantify the biomass in our dust samples, we scaled our bacterial ASV counts such that these bacterial counts and their relative abundances were comparable across samples despite the different durations of deployment.

Another limitation of this work is that we were unable to calculate the dust load mass in our passive dust collectors and were unable to measure the bacterial biomass in our dust samples. Future studies that quantify the dust load and dust bacterial biomass in Salton Sea dust will help to clarify the dispersal dynamics of bacteria in dust, and how these mechanisms vary amongst different taxa. Specifically, dust biomass, coupled with dust fractionation, could be used to determine which bacteria are found at different elevations after dust deposition, and how microbial dispersal, deposition, and diversity influence ecosystem dynamics. We were also unable to explore the microbial composition and functional diversity of Salton Sea soils. Characterizing the microbial diversity of the Salton Sea soils would elucidate the ecological importance of soil bacterial and dust bacteria, and how these communities interact during entrainment and after dust deposition. Investigating the Salton Sea soil microbiome in concert with the dust microbiome would also yield insight into which soil microorganisms are vulnerable to wind erosion and how these soil microorganisms colonize the atmospheric microbial community.

Lastly, we did not include a sampling negative control (i.e., a sterile passive dust collector) in our sequenced library pools, and thus, it is possible that some bacteria identified in this work were attributed to the passive dust collector itself rather than the Salton Sea dust. Studies investigating the bacterial diversity of low biomass samples have identified several taxa that were described in our work, including *Massilia, Sphingomonas*, and *Novosphinogobium* (Eisenhofer et al., 2019). However, we included sterile 0.2 um filters in the DNA extraction process and PCR amplification, and these negative controls were sequenced and analyzed with our samples to identify contaminants. As described in the Methods, we used the decontam package in R to remove contaminant ASVs from our samples, and we removed all ASVs found in our sequenced negative controls. We also removed all eukaryotic sequences that were identified in our 16S rRNA amplicon sequences. Though these steps can prevent contamination, future studies should include negative controls throughout the sampling process when dealing with low biomass samples (Fierer et al., 2025).

## Conclusion

We characterized the taxonomic and functional diversity of the aeolian dust microbiome from the Salton Sea ecosystem. The microbiome of the aeolian dust revealed that although there were location-specific differences in microbial composition between our four sites, there was a core microbiome of bacterial genera that were shared across the dust samples. The aeolian dust microbiome also contained a conserved set of genes that contribute to its survival in the atmospheric space, specifically genes involved in UV-radiation resistance, thermal resistance, and osmotic stress. Moreover, the conservation and redundancy of these functions in the aeolian dust microbiome appeared to be selected for by meteorological characteristics, such as relative humidity and precipitation, and contributing dust sources.

Our results help to establish a direct connection between microbial ecology in the environment and public health. The field of microbiology has focused on studying human pathogens, which is reflected by the lack of environmentally sourced and non-culturable microorganisms in our reference databases (Steen et al., 2019; Dias et al., 2020). The characterization of environmental microbiomes is crucial because as climate change worsens, our environment will continue to select for resilient and resistant microorganisms that may be pathogenic or can readily become pathogenic by receiving harmful traits via HGT. While the dust microorganisms we observed were not necessarily pathogenic, their adaptations allow them to remain viable across long distances, increasing the likelihood that they are inhaled upon exposure and induce inflammation and/or host microbiome dysbiosis. Furthermore, the Salton Sea aeolian microbiome contains multi-drug ARGs that can be transmitted via HGT to the host during inhalation and subsequent colonization of the airway and lungs, potentially reducing the efficacy of antibiotic treatment and thus increasing the vulnerability of the exposed populations to respiratory illness. Considering the high rate of respiratory distress and asthma experienced by the community in the Salton Sea region, the variety of ARGs and other potentially dangerous adaptations we found in the Salton Sea aeolian microbiome could pose a serious threat to the local population. Thus, understanding the accumulation and exchange of ARGs and other traits in the dust microbiome, and their transmission to host microbiomes via exposure and HGT, warrants further study. As saline lakes like the Salton Sea are shrinking globally and increasing the global dust load, it is plausible that this dust will drive the structure and function of global atmospheric dust microbiomes, as we observed in the dust of the Salton Sea region, and could have unforeseen, detrimental health impacts. We use this work to urge healthcare professionals, policy makers, and community members alike to consider the importance of environmental microbiomes, particularly dust microbiomes, as they work to combat the harm caused by water diversion and climate change.

## Data Availability

The datasets presented in this study can be found in online repositories. The names of the repository/repositories and accession number(s) can be found at: https://www.ncbi.nlm.nih.gov/, PRJNA1157902. The scripts used for the processing and analysis of these sequence data can be found at the following GitHub repository: https://github.com/hlfreund/SaltonSeaDustMicrobiology.
