# Supplementary material for: Weather conditions structure the taxonomic and functional diversity of the aeolian dust microbiome
Source: Front Microbiol. 2026 Mar 25;17:1691133. doi: 10.3389/fmicb.2026.1691133 (PMC13057538; doi:10.3389/fmicb.2026.1691133)
Supplement: Supplementary file 1 [file Data_Sheet_1.docx]

**Supplemental Figures and Tables**


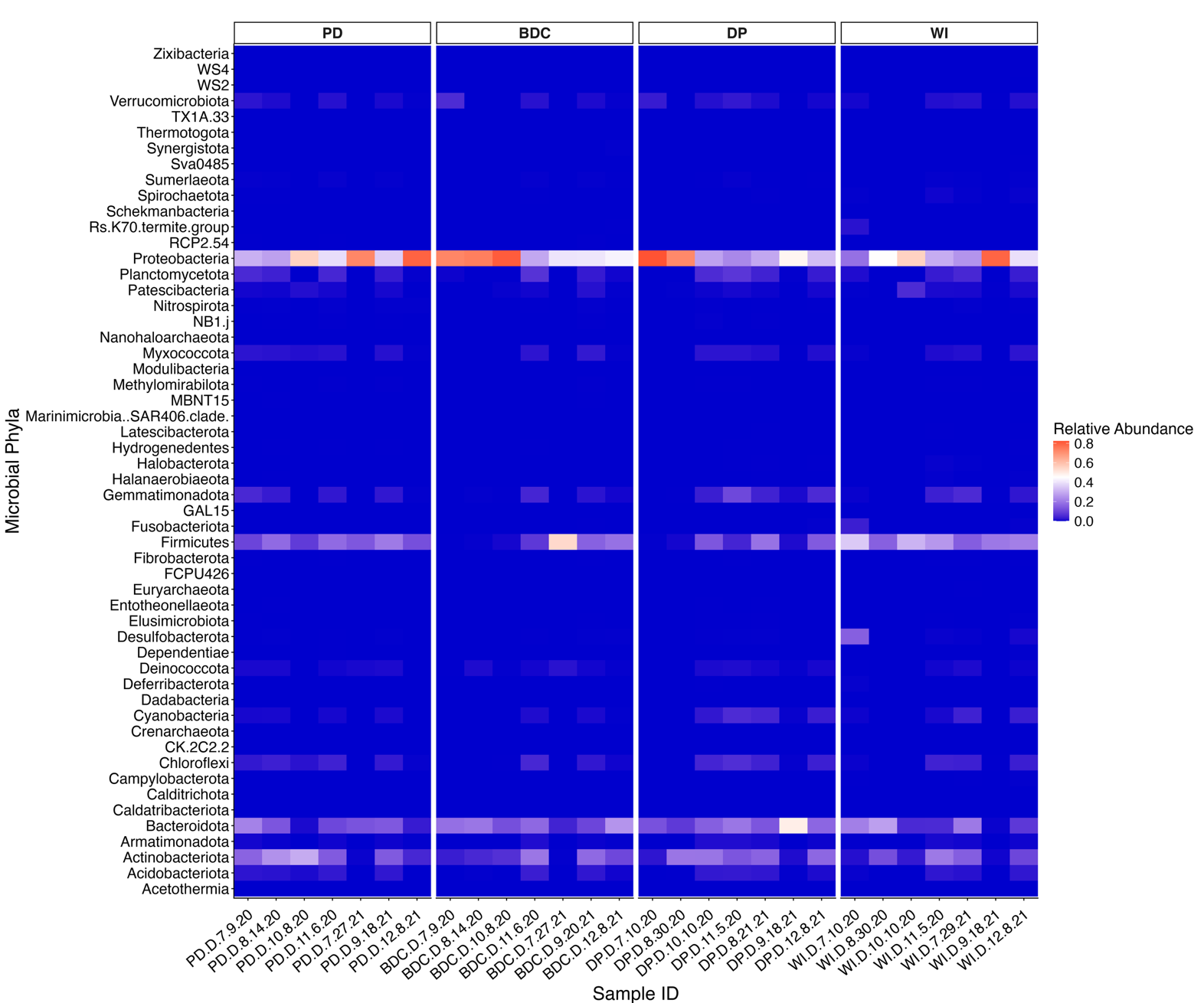


**Supplemental Figure 1. Bacterial Phyla Relative Abundance by Sample and Site.** This heatmap shows the relative abundance of bacterial phyla found in each sample and are separated by site: Palm Desert (PD), Boyd Deep Canyon (BDC), Dos Palmas (DP), and Wister (WI). Each column is a sample. The x-axis are sample IDs and the y-axis are bacterial phyla.

**
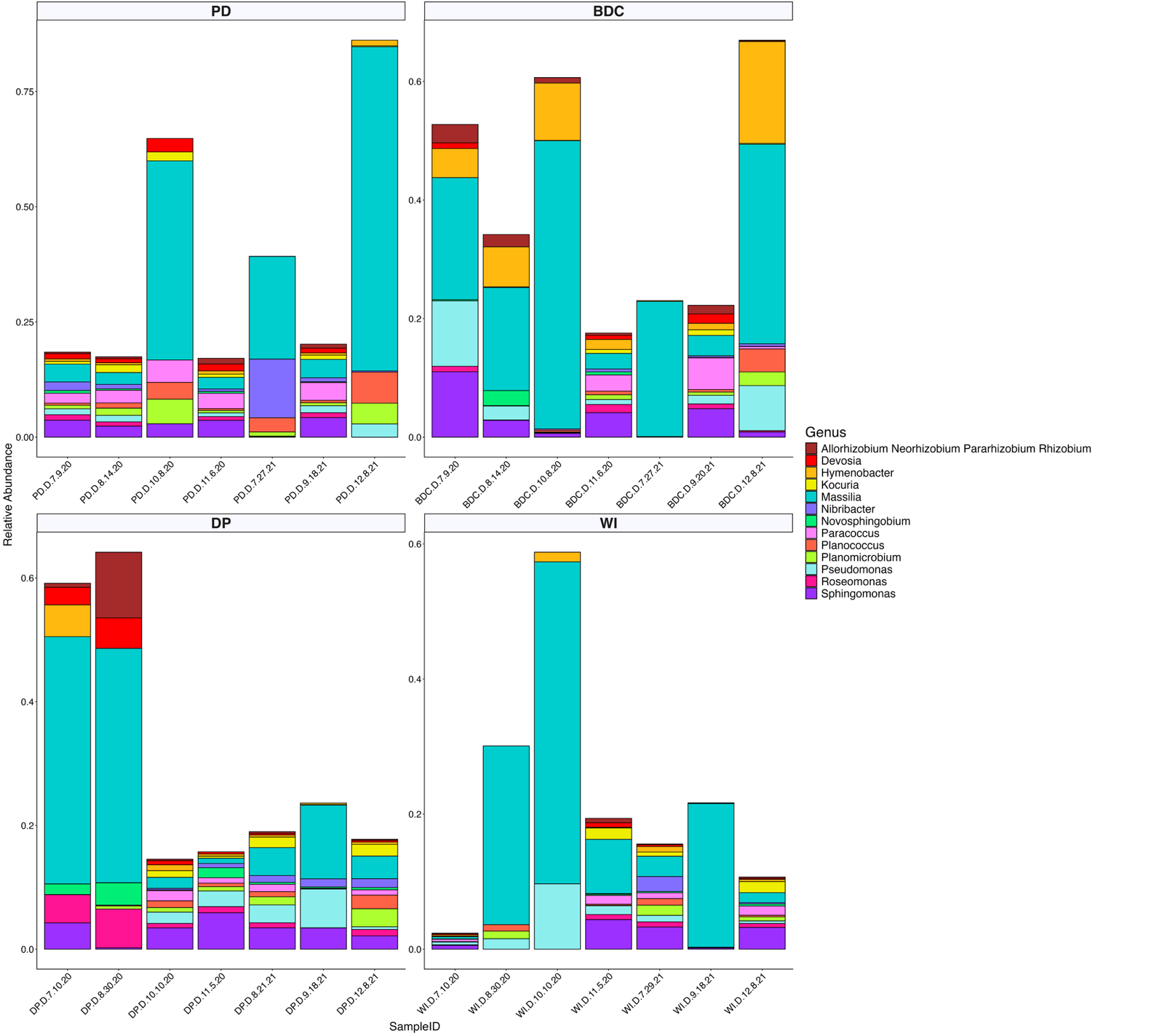
**

**Supplemental Figure 2. Core Microbiome Bacterial Genera by Sample and Site.** These stacked bar plots show the relative abundance of bacterial genera that were found in the core dust microbiome from the Salton Sea. Each column represents a sample, and samples are organized from left to right by collection date, then site (PD, BDC, DP, and WI).


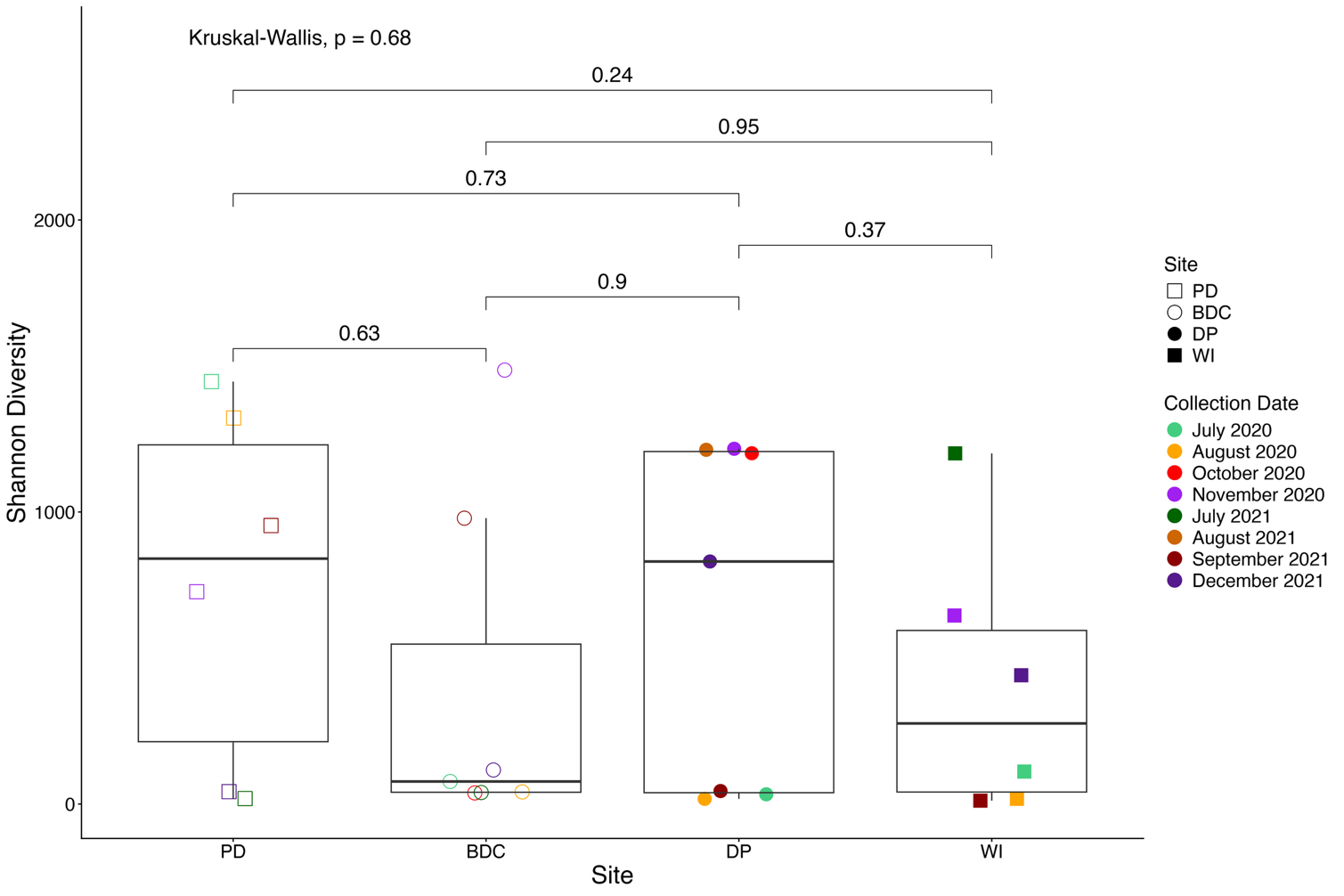


**Supplemental Figure 3. Shannon-Weiner Diversity by Site and Collection Date.** These box-and-whisker plots show the Shannon-Weiner diversity calculated from rarefied ASV counts from each sample within each site. The shape of the points indicate the site, whereas the color indicates the collection date.


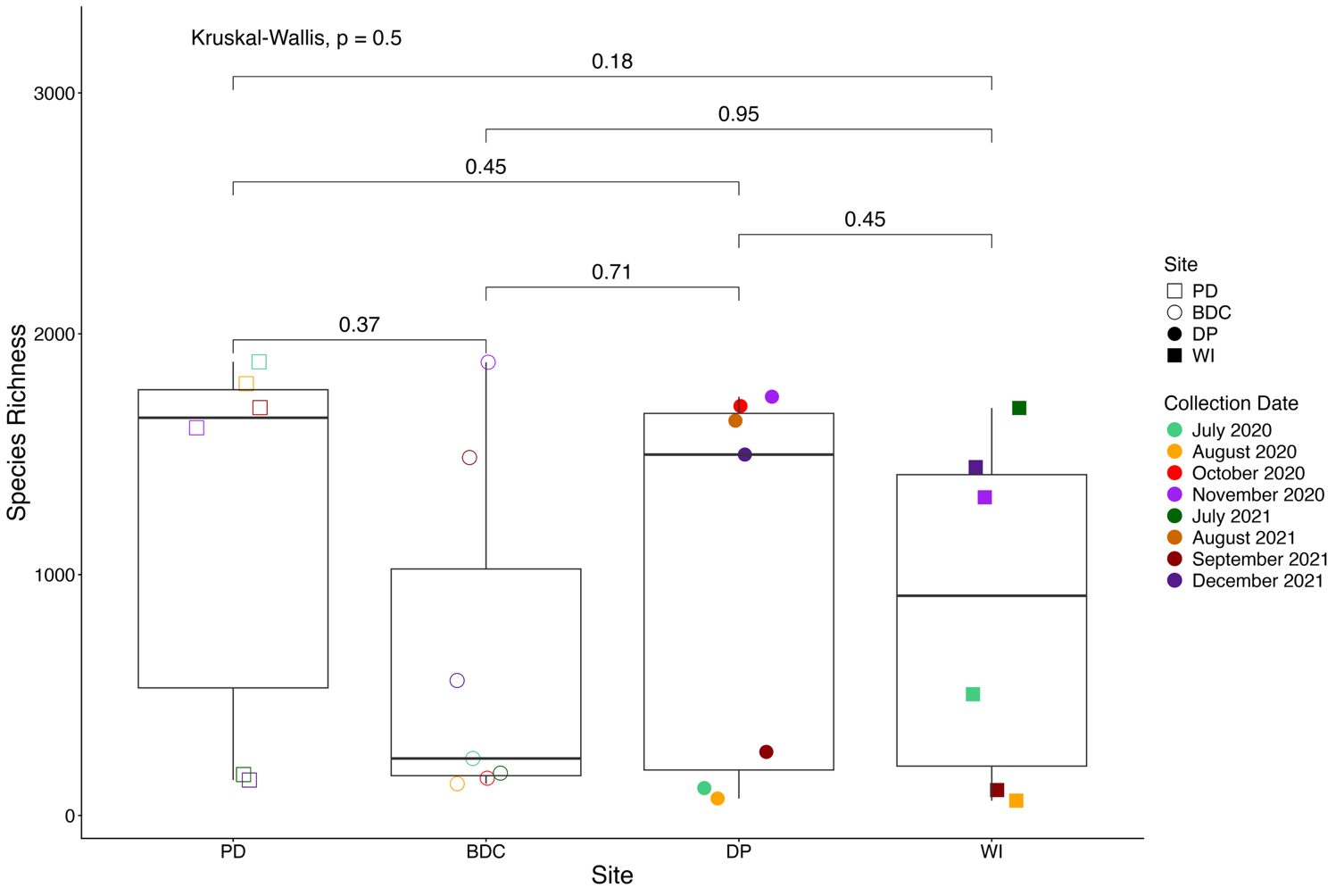


**Supplemental Figure 4. Species Richness by Site and Collection Date.** These box-and-whisker plots show the species richness calculated from rarefied ASV counts from each sample within each site. The shape of the points indicate the site, whereas the color indicates the collection date.

**
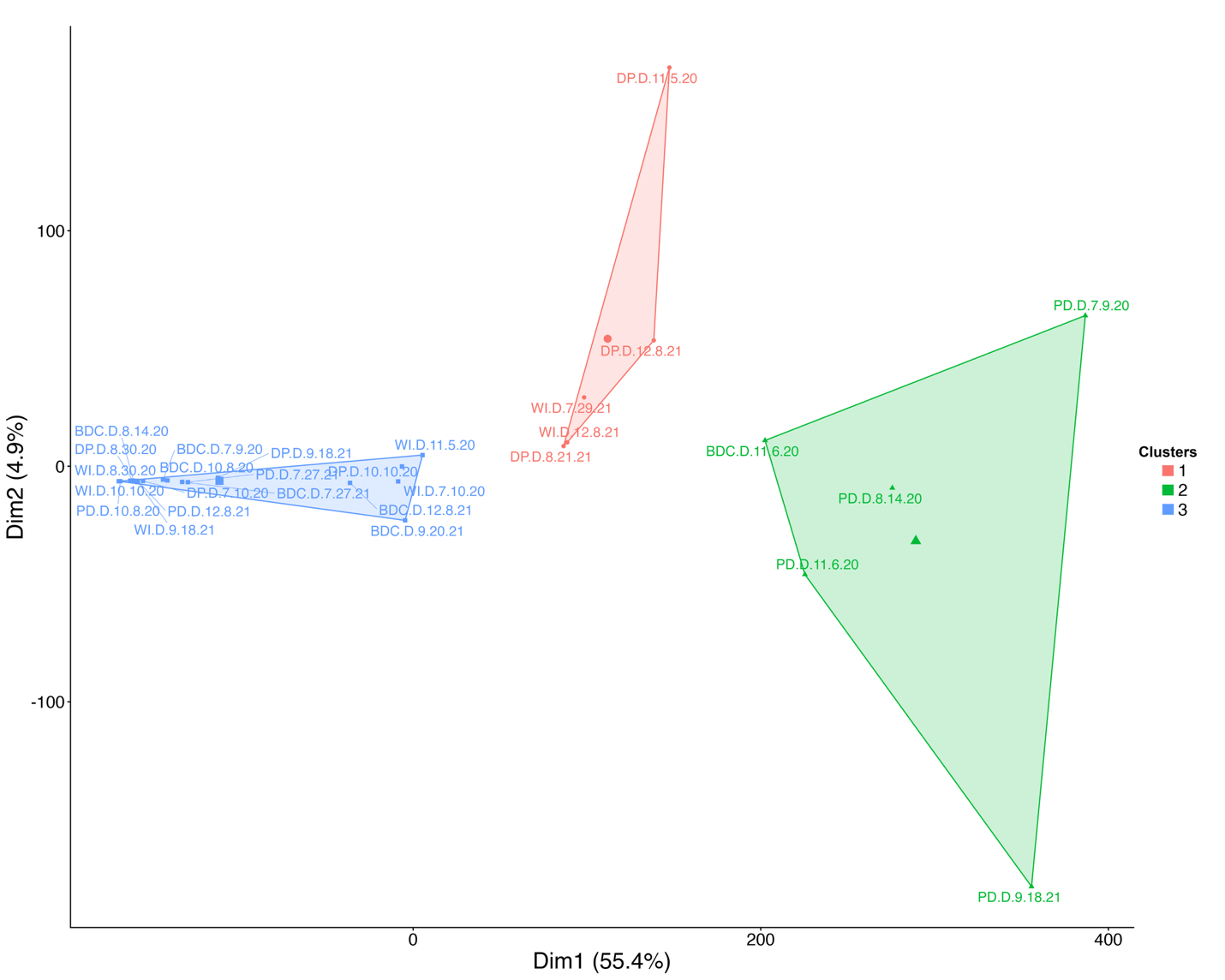
**

**Supplemental Figure 5. K-Means Clustering of Microbial Composition Data.** This is a

principle coordinates analysis (PCoA) showing the K-means clustering of the samples. Each point represents an individual samples, and all points within a cluster only belong

to that cluster. The clusters have been assigned the colors blue, red, and green.

**
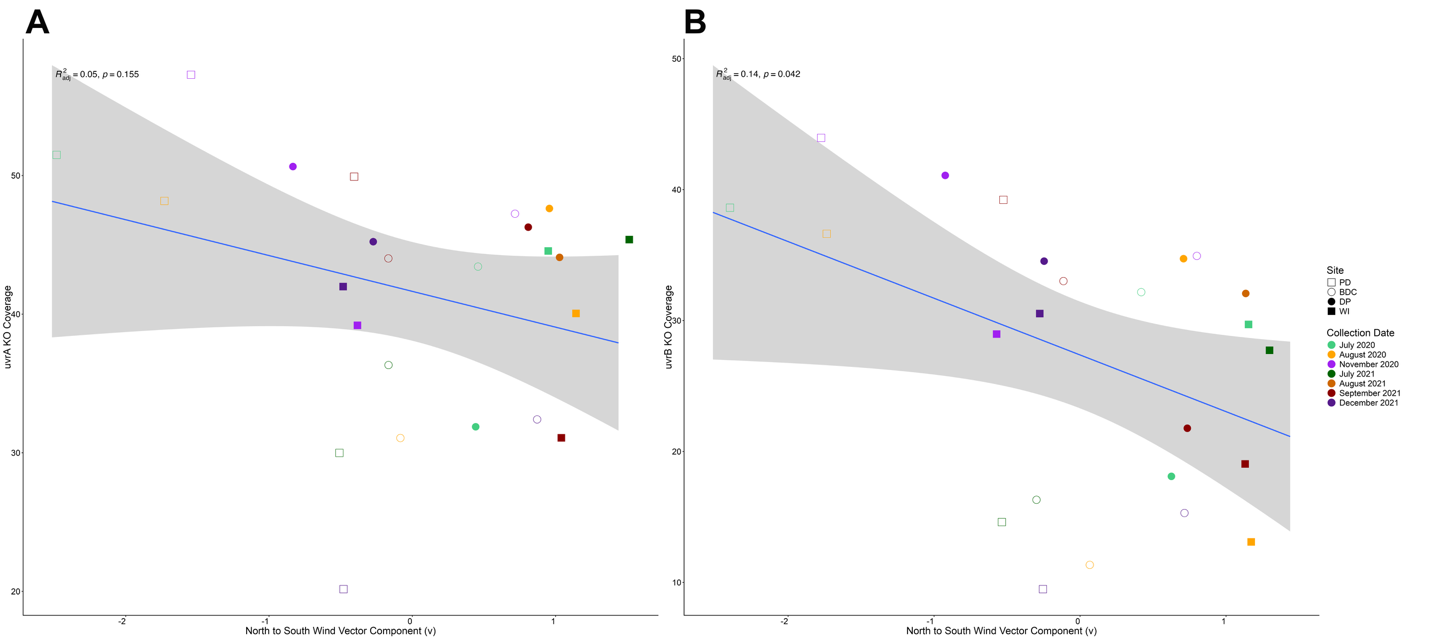
**

**Supplemental Figure 6. UV Radiation Resistance Genes *uvrA* (A) and *uvrB* (B), plotted against the Meridional Wind Component (north-south, v).** These scatterplots show how the normalized coverages of these genes decreased with the meridional wind component. The meridional wind component is on the x-axis, and the normalized coverage of the genes is on the y-axis. Points to the left of the center of the x-axis are predominantly northerly winds, whereas points to the right of the center are southerly winds.

**
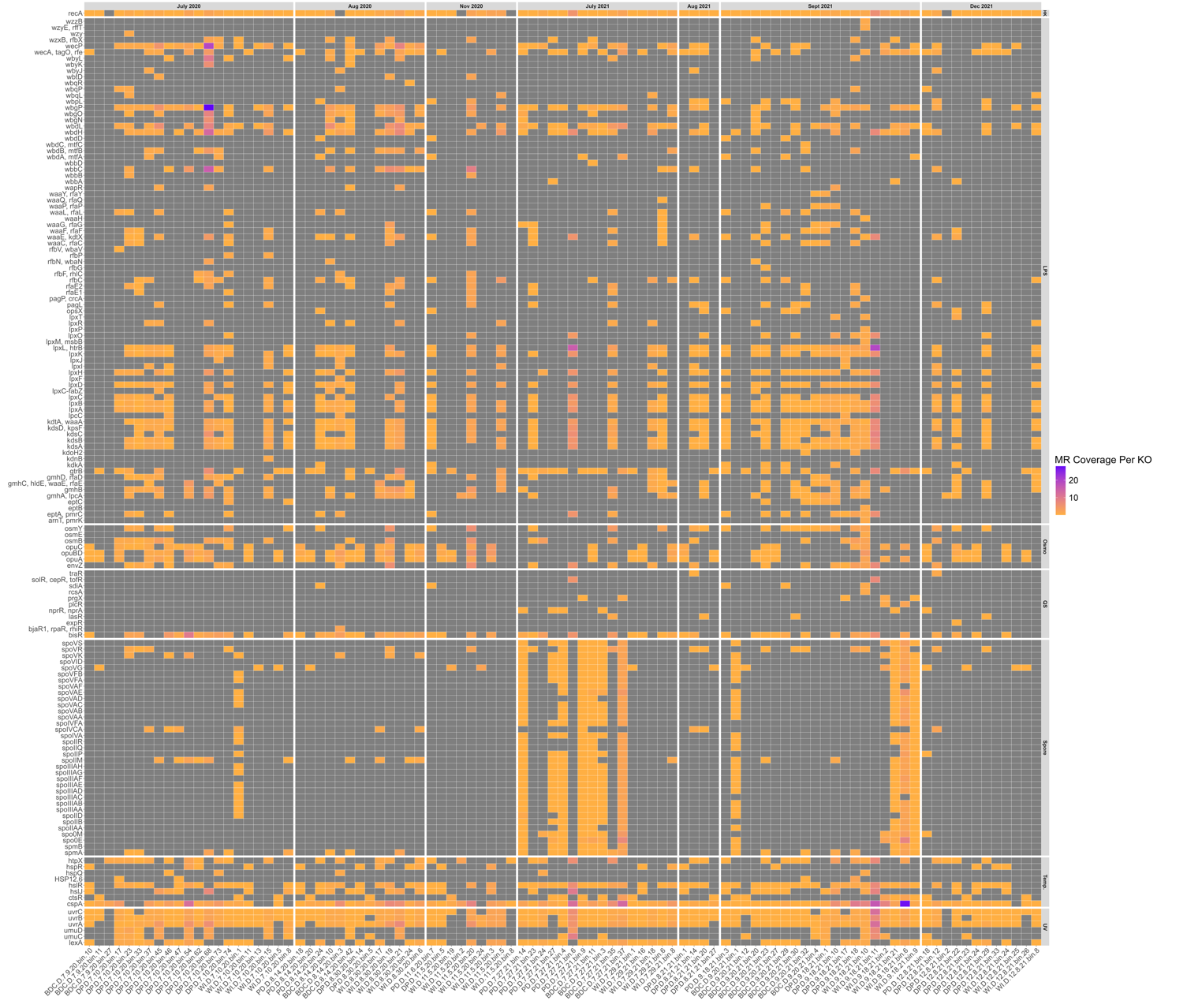
**

**Supplemental Figure 7. Heatmap of Atmospheric Survival Adaptations (Genes) and their Normalized Coverage in the Metagenome-Assembled Genomes (MAGs).** This heatmap shows the normalized coverage (median-ratio normalized, scaled coverages) of specific genes involved in surviving the atmospheric environment found in the MAGs. Each column represents a MAG, and the MAGs are organized by collection date from left to right. The genes are separated into functional categories: LPS modification genes (LPS), osmoprotectant transport/accumulation genes (Osmo), quorum sensing genes (QS), sporulation genes (Spore), temperature resistance genes (Temp), and UV radiation resistance genes (UV). Gray squares indicate that the gene is absent. The x-axis contains sample IDs, and the y-axis contains gene IDs for each KO.

**
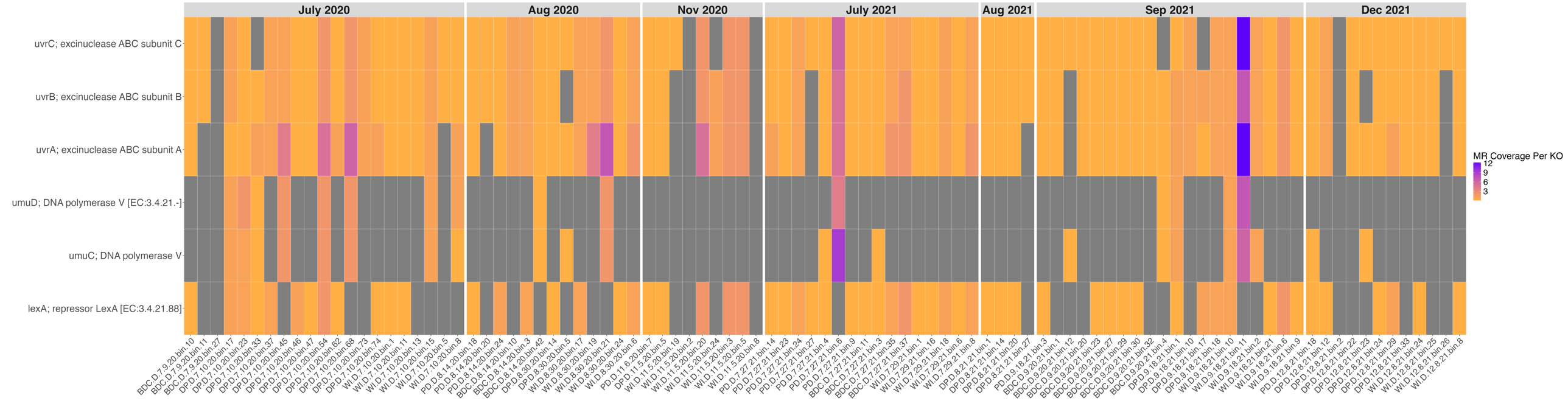
**

**Supplemental Figure 8. Heatmap of UV Radiation Resistance Genes in the Metagenome Assembled Genomes (MAGs).** This heatmap shows the normalized coverage (median-ratio normalized, scaled coverages) of UV radiation resistance genes in the MAGs. Each column represents a MAG, and the MAGs are organized by collection date from left to right. The y-axis contains UV radiation genes. Gray squares indicate that the gene is absent.

**
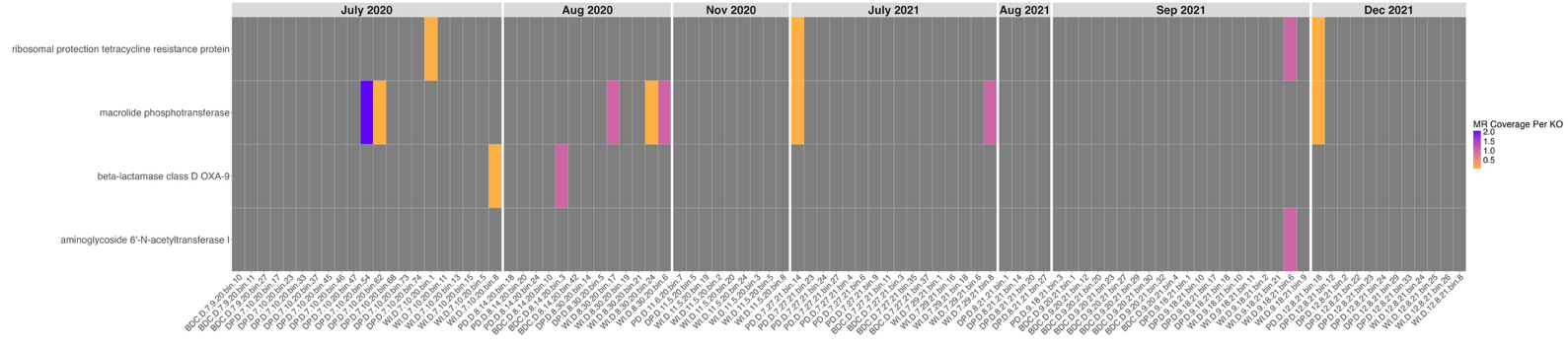
**

**Supplemental Figure 9. Heatmap of Antibiotic Resistance Genes in the Metagenome Assembled Genomes (MAGs).** This heatmap shows the normalized coverage (median-ratio normalized, scaled coverages) of antibiotic resistance genes (ARGs) in the MAGs. Each column represents a MAG, and the MAGs are organized by collection date from left to right. Gray squares indicate that the gene is absent.

| **Sample ID** | **Site** | **Collection Year** | **Sample Month** | **Deployment Date** | **Collection Date** | **Deployment Duration** | **Latitude** | **Longitude** |
| --- | --- | --- | --- | --- | --- | --- | --- | --- |
| PD.D.7.9.20 | PD | 2020 | July | 5/13/20 | 7/9/20 | 57 | 33.773808 | -116.35286 |
| PD.D.8.14.20 | PD | 2020 | August | 7/9/20 | 8/14/20 | 36 | 33.773808 | -116.35286 |
| PD.D.10.8.20 | PD | 2020 | October | 8/14/20 | 10/8/20 | 55 | 33.773808 | -116.35286 |
| PD.D.11.6.20 | PD | 2020 | November | 10/8/20 | 11/6/20 | 29 | 33.773808 | -116.35286 |
| PD.D.7.27.21 | PD | 2021 | July | 6/5/21 | 7/27/21 | 52 | 33.773808 | -116.35286 |
| PD.D.9.18.21 | PD | 2021 | September | 7/27/21 | 9/18/21 | 53 | 33.773808 | -116.35286 |
| PD.D.12.8.21 | PD | 2021 | December | 9/18/21 | 12/8/21 | 81 | 33.773808 | -116.35286 |
| BDC.D.7.9.20 | BDC | 2020 | July | 5/13/20 | 7/9/20 | 57 | 33.6516667 | -116.37264 |
| BDC.D.8.14.20 | BDC | 2020 | August | 7/9/20 | 8/14/20 | 36 | 33.6516667 | -116.37264 |
| BDC.D.10.8.20 | BDC | 2020 | October | 8/14/20 | 10/8/20 | 55 | 33.6516667 | -116.37264 |
| BDC.D.11.6.20 | BDC | 2020 | November | 10/8/20 | 11/6/20 | 29 | 33.6516667 | -116.37264 |
| BDC.D.7.27.21 | BDC | 2021 | July | 6/5/21 | 7/27/21 | 52 | 33.6516667 | -116.37264 |
| BDC.D.9.20.21 | BDC | 2021 | September | 7/27/21 | 9/20/21 | 55 | 33.6516667 | -116.37264 |
| BDC.D.12.8.21 | BDC | 2021 | December | 9/29/21 | 12/8/21 | 70 | 33.6516667 | -116.37264 |
| DP.D.7.10.20 | DP | 2020 | July | 6/1/20 | 7/10/20 | 39 | 33.48859 | -115.83517 |
| DP.D.8.30.20 | DP | 2020 | August | 7/10/20 | 8/30/20 | 51 | 33.48859 | -115.83517 |
| DP.D.10.10.20 | DP | 2020 | October | 8/30/20 | 10/10/20 | 41 | 33.48859 | -115.83517 |
| DP.D.11.5.20 | DP | 2020 | November | 10/10/20 | 11/5/20 | 26 | 33.48859 | -115.83517 |
| DP.D.8.21.21 | DP | 2021 | August | 6/8/21 | 8/19/21 | 72 | 33.48859 | -115.83517 |
| DP.D.9.18.21 | DP | 2021 | September | 8/19/21 | 9/18/21 | 30 | 33.48859 | -115.83517 |
| DP.D.12.8.21 | DP | 2021 | December | 9/18/21 | 12/8/21 | 81 | 33.48859 | -115.83517 |
| WI.D.7.10.20 | WI | 2020 | July | 6/1/20 | 7/10/20 | 39 | 33.283861 | -115.60008 |
| WI.D.8.30.20 | WI | 2020 | August | 7/10/20 | 8/30/20 | 51 | 33.283861 | -115.60008 |
| WI.D.10.10.20 | WI | 2020 | October | 8/30/20 | 10/10/20 | 41 | 33.283861 | -115.60008 |
| WI.D.11.5.20 | WI | 2020 | November | 10/10/20 | 11/5/20 | 26 | 33.283861 | -115.60008 |
| WI.D.7.29.21 | WI | 2021 | July | 6/8/21 | 7/29/21 | 51 | 33.283861 | -115.60008 |
| WI.D.9.18.21 | WI | 2021 | September | 7/29/21 | 9/18/21 | 51 | 33.283861 | -115.60008 |
| WI.D.12.8.21 | WI | 2021 | December | 9/18/21 | 12/8/21 | 81 | 33.283861 | -115.60008 |

**Supplemental Table 1. Sample Metadata.** This table details the metadata for each sample. PD represents Palm Desert, BDC represents Boyd Deep Canyon, DP represents Dos Palmas, and WI represents Wister.

| **Sample ID** | **Bin Num** | **Taxa Level** | **Marker Lineage** | **Lineage ID** | **Genome**  **Num** | **Completeness** | **Contamination** | **Strain**  **Heterogeneity** | **GC_Content** |
| --- | --- | --- | --- | --- | --- | --- | --- | --- | --- |
| DP_D_8_21_21_A | bin.1 | o | Actinomycetales | (UID1590) | 562 | 98.46 | 0.11 | 50 | 70.3 |
| DP_D_8_21_21_A | bin.14 | c | Alphaproteobacteria | (UID3305) | 564 | 98.69 | 4.41 | 7.14 | 67.5 |
| DP_D_8_21_21_A | bin.20 | c | Gammaproteobacteria | (UID4267) | 119 | 98.89 | 2.46 | 6.25 | 67.4 |
| DP_D_8_21_21_A | bin.27 | c | Bacilli | (UID285) | 586 | 92.53 | 0.72 | 50 | 50.7 |
| BDC_D_9_20_21_B | bin.1 | o | Bacillales | (UID828) | 139 | 89.66 | 0 | 0 | 38.6 |
| BDC_D_9_20_21_B | bin.12 | c | Bacilli | (UID285) | 586 | 83.48 | 0.72 | 50 | 51.2 |
| BDC_D_9_20_21_B | bin.20 | p | Bacteroidetes | (UID2605) | 350 | 86.27 | 1.32 | 100 | 40.7 |
| BDC_D_9_20_21_B | bin.23 | k | Bacteria | (UID203) | 5449 | 81.93 | 0.88 | 100 | 61.4 |
| BDC_D_9_20_21_B | bin.27 | o | Actinomycetales | (UID1590) | 562 | 97.27 | 0.06 | 0 | 70.3 |
| BDC_D_9_20_21_B | bin.29 | c | Gammaproteobacteria | (UID4202) | 67 | 98.55 | 0 | 0 | 61.9 |
| BDC_D_9_20_21_B | bin.30 | c | Gammaproteobacteria | (UID4267) | 119 | 98.54 | 2.29 | 7.69 | 67.6 |
| BDC_D_9_20_21_B | bin.32 | o | Burkholderiales | (UID4002) | 107 | 80.8 | 3.29 | 36.84 | 65.5 |
| BDC_D_9_20_21_B | bin.4 | c | Gammaproteobacteria | (UID4387) | 965 | 83.18 | 2.07 | 54.55 | 33.9 |
| WI_D_7_29_21_A | bin.1 | c | Bacilli | (UID285) | 586 | 93.1 | 0.72 | 50 | 50.7 |
| WI_D_7_29_21_A | bin.16 | o | Actinomycetales | (UID1590) | 562 | 99.06 | 0.7 | 25 | 70.3 |
| WI_D_7_29_21_A | bin.18 | o | Rhodospirillales | (UID3754) | 63 | 98.91 | 0 | 0 | 57.1 |
| WI_D_7_29_21_A | bin.6 | c | Gammaproteobacteria | (UID4201) | 1164 | 99.43 | 1.15 | 0 | 47.5 |
| WI_D_7_29_21_A | bin.8 | o | Actinomycetales | (UID1593) | 69 | 96.36 | 1.3 | 16.67 | 72.7 |
| PD_D_11_6_20_B | bin.7 | c | Gammaproteobacteria | (UID4202) | 67 | 98.55 | 0 | 0 | 61.9 |
| BDC_D_12_8_21_A | bins.30 | c | Bacilli | (UID259) | 750 | 96.52 | 0.99 | 50 | 43.3 |
| BDC_D_12_8_21_A | bins.6 | o | Clostridiales | (UID1212) | 172 | 87.25 | 0 | 0 | 48.8 |
| BDC_D_11_6_20_A | bins.11 | c | Alphaproteobacteria | (UID3305) | 564 | 85.92 | 3.86 | 87.5 | 45.2 |
| BDC_D_11_6_20_A | bins.15 | c | Bacilli | (UID285) | 586 | 89.94 | 0.72 | 50 | 50.7 |
| BDC_D_11_6_20_A | bins.20 | c | Gammaproteobacteria | (UID4202) | 67 | 98.55 | 0 | 0 | 61.9 |
| BDC_D_11_6_20_A | bins.25 | o | Actinomycetales | (UID1590) | 562 | 98.46 | 0.26 | 33.33 | 70.3 |
| BDC_D_8_14_20_A | bin.10 | p | Bacteroidetes | (UID2605) | 350 | 96.16 | 4.35 | 53.85 | 40.8 |
| BDC_D_8_14_20_A | bin.3 | o | Rhizobiales | (UID3447) | 356 | 98.39 | 1.21 | 0 | 66.2 |
| BDC_D_8_14_20_A | bin.42 | o | Cytophagales | (UID2936) | 47 | 98.21 | 0.6 | 0 | 52.5 |
| DP_D_8_30_20_A | bin.14 | o | Actinomycetales | (UID1590) | 562 | 98.46 | 0.82 | 16.67 | 70.3 |
| DP_D_8_30_20_A | bin.5 | c | Bacilli | (UID285) | 586 | 90.09 | 0.72 | 50 | 51.1 |
| DP_D_12_8_21_B | bin.12 | c | Alphaproteobacteria | (UID3305) | 564 | 98.69 | 4.41 | 7.14 | 67.5 |
| DP_D_12_8_21_B | bin.2 | k | Bacteria | (UID2495) | 2993 | 98.77 | 0.62 | 100 | 25 |
| DP_D_12_8_21_B | bin.22 | c | Gammaproteobacteria | (UID4444) | 263 | 90.84 | 2.92 | 12.5 | 54.5 |
| DP_D_12_8_21_B | bin.23 | c | Bacilli | (UID285) | 586 | 91.38 | 0.72 | 50 | 50.7 |
| DP_D_12_8_21_B | bin.24 | o | Actinomycetales | (UID1590) | 562 | 98.46 | 0.11 | 50 | 70.3 |
| DP_D_12_8_21_B | bin.29 | c | Gammaproteobacteria | (UID4267) | 119 | 98.54 | 2.64 | 7.14 | 67.5 |
| DP_D_12_8_21_B | bin.33 | o | Actinomycetales | (UID1530) | 622 | 95.86 | 2.96 | 83.33 | 60.2 |
| BDC_D_7_9_20_A | bin.10 | o | Actinomycetales | (UID1590) | 562 | 99.06 | 0.11 | 50 | 70.3 |
| BDC_D_7_9_20_A | bin.11 | c | Bacilli | (UID285) | 586 | 92.53 | 0.72 | 50 | 50.7 |
| BDC_D_7_9_20_A | bin.27 | k | Bacteria | (UID2495) | 2993 | 97.18 | 0 | 0 | 23.2 |
| DP_D_7_10_20_C | bin.17 | o | Rhizobiales | (UID3447) | 356 | 96.43 | 1.02 | 0 | 61 |
| DP_D_7_10_20_C | bin.23 | o | Burkholderiales | (UID4000) | 193 | 92.4 | 4.19 | 15.38 | 68 |
| DP_D_7_10_20_C | bin.33 | o | Burkholderiales | (UID4000) | 193 | 89.68 | 4.63 | 56.25 | 65.4 |
| DP_D_7_10_20_C | bin.37 | o | Rhodospirillales | (UID3754) | 63 | 90.69 | 3.61 | 7.69 | 69.5 |
| DP_D_7_10_20_C | bin.45 | o | Cytophagales | (UID2936) | 47 | 95.89 | 1.34 | 20 | 61.3 |
| DP_D_7_10_20_C | bin.46 | k | Bacteria | (UID203) | 5449 | 80.34 | 0 | 0 | 68.4 |
| DP_D_7_10_20_C | bin.47 | o | Sphingomonadales | (UID3310) | 26 | 97.78 | 0.46 | 75 | 65.9 |
| DP_D_7_10_20_C | bin.54 | f | Micrococcaceae | (UID1631) | 31 | 95.03 | 0.55 | 25 | 65.9 |
| DP_D_7_10_20_C | bin.62 | o | Actinomycetales | (UID1593) | 69 | 93.98 | 2.53 | 0 | 67.8 |
| DP_D_7_10_20_C | bin.68 | o | Cytophagales | (UID2936) | 47 | 99.7 | 0.6 | 0 | 52.5 |
| DP_D_7_10_20_C | bin.73 | c | Alphaproteobacteria | (UID3422) | 26 | 83.32 | 3.29 | 82.35 | 66.1 |
| DP_D_7_10_20_C | bin.74 | p | Proteobacteria | (UID3887) | 1487 | 97.56 | 1.98 | 25 | 64.6 |
| WI_D_8_30_20_B | bin.17 | o | Actinomycetales | (UID1593) | 69 | 97.38 | 2.95 | 18.18 | 67.2 |
| WI_D_8_30_20_B | bin.19 | g | Burkholderia | (UID4006) | 64 | 92.68 | 2.98 | 42.86 | 59.3 |
| WI_D_8_30_20_B | bin.21 | o | Cytophagales | (UID2936) | 47 | 90.18 | 0.3 | 100 | 45.9 |
| WI_D_8_30_20_B | bin.24 | o | Actinomycetales | (UID1593) | 69 | 94.9 | 2.98 | 36.36 | 68.5 |
| WI_D_8_30_20_B | bin.6 | o | Actinomycetales | (UID1593) | 69 | 99.49 | 4.25 | 31.25 | 64.1 |
| PD_D_9_18_21_C | bin.3 | c | Gammaproteobacteria | (UID4202) | 67 | 98.55 | 0 | 0 | 61.9 |
| DP_D_11_5_20_A | bin.5 | o | Actinomycetales | (UID1590) | 562 | 97.87 | 1.4 | 0 | 70.3 |
| BDC_D_7_27_21_A | bin.11 | k | Bacteria | (UID203) | 5449 | 82.14 | 0.89 | 0 | 45.3 |
| BDC_D_7_27_21_A | bin.3 | f | Bacillaceae | (UID829) | 128 | 80.59 | 3.28 | 51.72 | 42.2 |
| BDC_D_7_27_21_A | bin.35 | o | Burkholderiales | (UID4000) | 193 | 92.12 | 2.44 | 33.33 | 65.7 |
| BDC_D_7_27_21_A | bin.37 | o | Bacillales | (UID828) | 139 | 89.63 | 1.6 | 0 | 49.1 |
| PD_D_12_8_21_A | bin.18 | c | Bacilli | (UID259) | 750 | 96.52 | 0.99 | 50 | 43.3 |
| DP_D_9_8_21_B | bin.1 | c | Gammaproteobacteria | (UID4387) | 965 | 96.74 | 0.14 | 100 | 34.3 |
| DP_D_9_8_21_B | bin.10 | c | Gammaproteobacteria | (UID4444) | 263 | 95.98 | 0.52 | 0 | 35.7 |
| DP_D_9_8_21_B | bin.17 | c | Alphaproteobacteria | (UID3305) | 564 | 82.35 | 2.53 | 81.82 | 45.2 |
| DP_D_9_8_21_B | bin.18 | o | Burkholderiales | (UID4002) | 107 | 94.74 | 2.5 | 13.64 | 62.6 |
| WI_D_9_18_21_A | bin.10 | f | Enterobacteriaceae | (UID5124) | 134 | 96.68 | 2.16 | 55.17 | 55.3 |
| WI_D_9_18_21_A | bin.11 | f | Moraxellaceae | (UID4680) | 86 | 95.39 | 0.27 | 0 | 39 |
| WI_D_9_18_21_A | bin.2 | c | Bacilli | (UID259) | 750 | 96.05 | 1.32 | 100 | 47.4 |
| WI_D_9_18_21_A | bin.21 | o | Bacillales | (UID828) | 139 | 91.11 | 1.99 | 12.5 | 49.6 |
| WI_D_9_18_21_A | bin.6 | o | Bacillales | (UID828) | 139 | 98.28 | 0.03 | 0 | 38.4 |
| WI_D_9_18_21_A | bin.9 | o | Bacillales | (UID828) | 139 | 96.52 | 1.73 | 0 | 48.8 |
| WI_D_12_8_21_A | bin.24 | o | Actinomycetales | (UID1590) | 562 | 98.46 | 0.11 | 50 | 70.3 |
| WI_D_12_8_21_A | bin.25 | c | Spirochaetia | (UID2496) | 72 | 98.13 | 0 | 0 | 27.3 |
| WI_D_12_8_21_A | bin.26 | c | Bacilli | (UID285) | 586 | 92.53 | 0.72 | 50 | 50.7 |
| WI_D_12_8_21_A | bin.8 | o | Rhodospirillales | (UID3754) | 63 | 98.91 | 0 | 0 | 57.1 |
| PD_D_8_14_20_B | bin.18 | o | Actinomycetales | (UID1590) | 562 | 98.46 | 0.11 | 50 | 70.3 |
| PD_D_8_14_20_B | bin.20 | c | Bacilli | (UID285) | 586 | 92.53 | 0.72 | 50 | 50.7 |
| PD_D_8_14_20_B | bin.24 | c | Gammaproteobacteria | (UID4202) | 67 | 98.55 | 0 | 0 | 61.9 |
| WI_D_7_10_20_A | bin.1 | o | Clostridiales | (UID1212) | 172 | 90.94 | 0 | 0 | 43.3 |
| WI_D_7_10_20_A | bin.11 | o | Actinomycetales | (UID1590) | 562 | 98.46 | 0.11 | 50 | 70.3 |
| WI_D_7_10_20_A | bin.13 | c | Spirochaetia | (UID2496) | 72 | 98.13 | 0 | 0 | 27.5 |
| WI_D_7_10_20_A | bin.15 | c | Deltaproteobacteria | (UID3218) | 61 | 97.04 | 0.59 | 100 | 45.5 |
| WI_D_7_10_20_A | bin.5 | c | Bacilli | (UID285) | 586 | 92.53 | 0.72 | 50 | 50.7 |
| WI_D_7_10_20_A | bin.8 | o | Bacteroidales | (UID2621) | 198 | 94.04 | 0.96 | 0 | 37.1 |
| WI_D_11_5_20_A | bin.19 | c | Bacilli | (UID285) | 586 | 91.38 | 0.72 | 50 | 50.7 |
| WI_D_11_5_20_A | bin.2 | k | Bacteria | (UID2495) | 2993 | 98.77 | 0.62 | 100 | 25 |
| WI_D_11_5_20_A | bin.20 | o | Burkholderiales | (UID4001) | 108 | 96.88 | 4.07 | 10 | 66.3 |
| WI_D_11_5_20_A | bin.24 | c | Spirochaetia | (UID2496) | 72 | 88 | 0 | 0 | 27.3 |
| WI_D_11_5_20_A | bin.3 | o | Actinomycetales | (UID1590) | 562 | 98.46 | 0.11 | 50 | 70.3 |
| WI_D_11_5_20_A | bin.5 | o | Rhodospirillales | (UID3754) | 63 | 98.91 | 0 | 0 | 57.1 |
| WI_D_11_5_20_A | bin.8 | k | Bacteria | (UID2495) | 2993 | 97.18 | 0 | 0 | 23.2 |
| PD_D_7_27_21_A | bin.14 | k | Bacteria | (UID203) | 5449 | 81.03 | 1.72 | 0 | 52.3 |
| PD_D_7_27_21_A | bin.23 | p | Proteobacteria | (UID3887) | 1487 | 82.61 | 0.62 | 0 | 71.6 |
| PD_D_7_27_21_A | bin.24 | c | Alphaproteobacteria | (UID3305) | 564 | 99.57 | 0.04 | 0 | 63.9 |
| PD_D_7_27_21_A | bin.27 | o | Bacillales | (UID828) | 139 | 81.34 | 1.99 | 12.5 | 49.8 |
| PD_D_7_27_21_A | bin.4 | f | Bacillaceae | (UID829) | 128 | 82.49 | 2.73 | 53.57 | 42.2 |
| PD_D_7_27_21_A | bin.6 | f | Moraxellaceae | (UID4680) | 86 | 93.74 | 0 | 0 | 38.8 |
| PD_D_7_27_21_A | bin.9 | o | Bacillales | (UID828) | 139 | 94.23 | 1.64 | 0 | 49 |

**Supplemental Table 2. CheckM Results for Metagenome-Assembled Genomes (MAGs) Bin Assignments**. This table contains the CheckM results for each putative, high-quality MAG bin assignment.

**Supplemental Table 3. Surface Type Frequencies.** This table contains the surface type frequencies for each sample. The categories for the surface types are Barren Land, Crop Land, Developed, Forest, Herbaceous, Mexico, Open Water, Others (i.e., not within surfaces listed), Salton Sea, and Shrub. The surface type frequencies for each sample total to one.

| **SampleID** | **Date**  **Start** | **Date**  **End** | **Barren**  **Land** | **Crop**  **Land** | **Developed** | **Forest** | **Herbaceous** | **Mexico** | **Open**  **Water** | **Others** | **Salton**  **Sea** | **Shrub** |
| --- | --- | --- | --- | --- | --- | --- | --- | --- | --- | --- | --- | --- |
| BDC.D.7.9.20 | 5/13/20 | 7/10/20 | 0.07322056 | 0.01297334 | 0.14651081 | 0.02503976 | 0.06772567 | 0.00604489 | 0.016034 | 0.01004535 | 2.69e-06 | 0.64240294 |
| BDC.D.8.14.20 | 7/10/20 | 8/30/20 | 0.02367024 | 0.0146429 | 0.12042544 | 0.032043 | 0.07802577 | 0.00198004 | 0.01920307 | 0.014634 | 0.00019225 | 0.6951833 |
| BDC.D.10.8.20 | 8/30/20 | 10/10/20 | 0.13443645 | 0.01089365 | 0.13340866 | 0.01440556 | 0.04611422 | 5.8e-05 | 0.00294073 | 0.00475144 | 0.00052367 | 0.65246762 |
| BDC.D.11.6.20 | 10/10/20 | 11/6/20 | 0.18093467 | 0.00680144 | 0.10053623 | 0.00868282 | 0.04070882 | 0 | 0.00103723 | 0.00198321 | 6.65e-05 | 0.65924912 |
| BDC.D.7.27.21 | 6/5/21 | 8/19/21 | 0.0681913 | 0.01551662 | 0.11158094 | 0.03471532 | 0.07214976 | 0.00440204 | 0.00669423 | 0.01092161 | 0.00048259 | 0.6753456 |
| BDC.D.9.20.21 | 8/19/21 | 10/1/21 | 0.16206552 | 0.02308375 | 0.09404165 | 0.02603989 | 0.05451374 | 0.01118828 | 0.00791524 | 0.00963109 | 0.00106754 | 0.61045329 |
| BDC.D.12.8.21 | 10/1/21 | 12/8/21 | 0.12686537 | 0.0135476 | 0.13669878 | 0.01164132 | 0.04738955 | 0 | 0.01735052 | 0.00527006 | 4.74e-05 | 0.64118935 |
| DP.D.7.10.20 | 5/13/20 | 7/10/20 | 0.3425539 | 0.03768778 | 0.05103636 | 0.0052857 | 0.02849791 | 0.01015245 | 0.00231048 | 0.00759165 | 0.08074333 | 0.43414044 |
| DP.D.8.30.20 | 7/10/20 | 8/30/20 | 0.35894683 | 0.04741788 | 0.05094149 | 0.00539539 | 0.02106017 | 0.00365814 | 0.00221514 | 0.00818428 | 0.08859748 | 0.4135832 |
| DP.D.10.10.20 | 8/30/20 | 10/10/20 | 0.30053338 | 0.02469771 | 0.03382912 | 0.00265595 | 0.04263803 | 0.00132635 | 0.0003168 | 0.00746317 | 0.01244322 | 0.57409628 |
| DP.D.11.5.20 | 10/10/20 | 11/6/20 | 0.18618336 | 0.00928874 | 0.02733272 | 0.00324026 | 0.06784859 | 4.4e-06 | 0.00052345 | 0.00358963 | 0.00123141 | 0.70075744 |
| DP.D.8.21.21 | 6/5/21 | 8/19/21 | 0.38694083 | 0.05133939 | 0.05987361 | 0.00382841 | 0.02037435 | 0.00892055 | 0.00069019 | 0.00823634 | 0.0924024 | 0.36739392 |
| DP.D.9.18.21 | 8/19/21 | 10/1/21 | 0.34635375 | 0.06346146 | 0.03868698 | 0.00385657 | 0.02248277 | 0.01872453 | 0.00038984 | 0.01222721 | 0.14363171 | 0.35018519 |
| DP.D.12.8.21 | 10/1/21 | 12/8/21 | 0.19903967 | 0.02407875 | 0.04713999 | 0.00413071 | 0.05948762 | 0.00133103 | 0.00736264 | 0.00678254 | 0.00963938 | 0.64100769 |
| PD.D.7.9.20 | 5/13/20 | 7/10/20 | 0.10861349 | 0.01199928 | 0.30194873 | 0.01816956 | 0.05927331 | 0.00299202 | 0.00974925 | 0.00803661 | 1.82e-06 | 0.47921594 |
| PD.D.8.14.20 | 7/10/20 | 8/30/20 | 0.05853381 | 0.01623364 | 0.36434508 | 0.02079035 | 0.07135971 | 0.00070631 | 0.0107935 | 0.00828889 | 6.45e-05 | 0.44888419 |
| PD.D.10.8.20 | 8/30/20 | 10/10/20 | 0.17780306 | 0.00940879 | 0.18829575 | 0.00962776 | 0.04294059 | 0 | 0.0013125 | 0.00345215 | 0.00036642 | 0.56679299 |
| PD.D.11.6.20 | 10/10/20 | 11/6/20 | 0.20801019 | 0.00330571 | 0.09345096 | 0.00801725 | 0.04145163 | 0 | 0.00081306 | 0.00132809 | 5.42e-06 | 0.64361768 |
| PD.D.7.27.21 | 6/5/21 | 8/19/21 | 0.09970635 | 0.01665161 | 0.34421231 | 0.0193936 | 0.05673494 | 0.00167123 | 0.00455538 | 0.00706927 | 0.0009234 | 0.44908191 |
| PD.D.9.18.21 | 8/19/21 | 10/1/21 | 0.13978181 | 0.02860087 | 0.3145067 | 0.017189 | 0.05098705 | 0.00471429 | 0.00417088 | 0.00765748 | 0.00837097 | 0.42402096 |
| PD.D.12.8.21 | 10/1/21 | 12/8/21 | 0.18101446 | 0.00704376 | 0.16126502 | 0.00887213 | 0.04736612 | 0 | 0.01273217 | 0.00346016 | 1.47e-05 | 0.57823151 |
| WI.D.7.10.20 | 5/13/20 | 7/10/20 | 0.45672618 | 0.05665537 | 0.03012024 | 0.0022247 | 0.02777861 | 0.02719231 | 0.00126884 | 0.01274943 | 0.14920259 | 0.23608173 |
| WI.D.8.30.20 | 7/10/20 | 8/30/20 | 0.50702475 | 0.05586727 | 0.02779496 | 0.002216 | 0.01756764 | 0.01832024 | 0.00085284 | 0.01389667 | 0.18704336 | 0.16941627 |
| WI.D.10.10.20 | 8/30/20 | 10/10/20 | 0.37051278 | 0.06611746 | 0.02867629 | 0.00135519 | 0.05782299 | 0.00933686 | 0.00023521 | 0.01407066 | 0.03993598 | 0.41193659 |
| WI.D.11.5.20 | 10/10/20 | 11/6/20 | 0.22655944 | 0.02693476 | 0.01637446 | 0.00065457 | 0.09026512 | 0.00052604 | 0.00184687 | 0.00462337 | 0.00323696 | 0.62897841 |
| WI.D.7.29.21 | 6/5/21 | 8/19/21 | 0.42048804 | 0.09138852 | 0.03429356 | 0.00201323 | 0.02025885 | 0.04146066 | 0.00041462 | 0.01784795 | 0.19155439 | 0.18028019 |
| WI.D.9.18.21 | 8/19/21 | 10/1/21 | 0.39852806 | 0.1301901 | 0.02982851 | 0.00137064 | 0.02558595 | 0.0519148 | 0.0005992 | 0.02287856 | 0.15902737 | 0.1800768 |
| WI.D.12.8.21 | 10/1/21 | 12/8/21 | 0.29890757 | 0.04348993 | 0.03635549 | 0.00276938 | 0.06039352 | 0.00550029 | 0.00628978 | 0.00802298 | 0.0310373 | 0.50723375 |

**Supplemental Table 4. Sample Climate and Precipitation Data.** This table contains the wind condition data and precipitation data from Synoptic. The precipitation data includes the average, accumulated precipitation (in a 24-hour period). The wind data includes average air temperature, average wind speed, average relative humidity, the average zonal wind component (east-west, u), and meridional wind component (north-south, v).

| **SampleID** | **STID** | **Precip.STID** | **Deploy Date** | **Collect Date** | **Ave. Accum. Precip (24hr)** | **Ave. Air Temp** | **Ave. Wind Speed** | **Ave. Relative Humidity** | **Ave. Wind Component u (E-W)** | **Ave. Wind Component v (N-S)** |
| --- | --- | --- | --- | --- | --- | --- | --- | --- | --- | --- |
| PD.D.7.9.20 | CI200 | C2285 | 5/13/20 | 7/9/20 | 0 | 31.6192405 | 3.17936125 | 30.5940233 | 1.44284857 | -1.7500013 |
| WI.D.7.10.20 | CQ125 | D3583 | 6/1/20 | 7/10/20 | 0 | 31.4917673 | 3.20078266 | 27.5925357 | 0.21191109 | 1.1614761 |
| DP.D.7.10.20 | DPMC1 | COOPMCAC1 | 6/1/20 | 7/10/20 | 0 | 32.363759 | 2.11469002 | 24.6093418 | 0.33744625 | 0.72253833 |
| BDC.D.7.9.20 | UCDE | COOPDEEC1 | 5/13/20 | 7/9/20 | 0 | 29.800629 | 2.89061972 | 22.0888646 | 0.10022889 | 0.65080543 |
| PD.D.8.14.20 | CI200 | C2285 | 7/9/20 | 8/14/20 | 0 | 36.6467379 | 2.87533448 | 26.9252874 | 1.08484132 | -1.0977327 |
| WI.D.8.30.20 | CQ125 | D3583 | 7/10/20 | 8/30/20 | 0 | 35.6510586 | 2.69738355 | 31.4058632 | -0.5215483 | 1.21374693 |
| DP.D.8.30.20 | DPMC1 | COOPMCAC1 | 7/10/20 | 8/30/20 | 0 | 36.3095279 | 1.88516995 | 28.3029557 | 0.15674952 | 1.03202101 |
| BDC.D.8.14.20 | UCDE | COOPDEEC1 | 7/9/20 | 8/14/20 | 0 | 34.584064 | 2.40315613 | 17.6465517 | -0.3452522 | 0.35372597 |
| PD.D.10.8.20 | CI200 | C2285 | 8/14/20 | 10/8/20 | 0 | 32.7084236 | 2.19324196 | 33.6508058 | 0.62860594 | -0.3656956 |
| WI.D.10.10.20 | CQ125 | D3583 | 8/30/20 | 10/10/20 | 0 | 30.9193939 | 2.23787677 | 31.9631313 | -0.8053231 | 0.41322402 |
| DP.D.10.10.20 | DPMC1 | COOPMCAC1 | 8/30/20 | 10/10/20 | 0 | 31.9323771 | 1.49573407 | 24.5995956 | 0.11249049 | 0.45022024 |
| BDC.D.10.8.20 | UCDE | COOPDEEC1 | 8/14/20 | 10/8/20 | 0 | 34.3467205 | 2.48082334 | 17.7515083 | 0.11591291 | 0.65468414 |
| PD.D.11.6.20 | CI200 | C2285 | 10/8/20 | 11/6/20 | 0 | 24.3785977 | 2.27683595 | 37.6091298 | 1.07231029 | -1.062306 |
| WI.D.11.5.20 | CQ125 | D3583 | 10/10/20 | 11/5/20 | 0 | 23.5542265 | 2.4398756 | 36.100319 | -0.1189558 | -0.0414189 |
| DP.D.11.5.20 | DPMC1 | COOPMCAC1 | 10/10/20 | 11/5/20 | 0 | 25.0017599 | 1.63676312 | 25.0063593 | 0.58507038 | -0.3522882 |
| BDC.D.11.6.20 | UCDE | COOPDEEC1 | 10/8/20 | 11/6/20 | 0 | 29.8421252 | 2.58693316 | 19.1742809 | 0.77225557 | 0.86968527 |
| PD.D.7.27.21 | CI200 | C2285 | 6/5/21 | 7/27/21 | 0.22632446 | 35.177882 | 2.92311164 | 29.6523126 | 0.97276723 | -0.0770987 |
| WI.D.7.29.21 | CQ125 | D3583 | 6/8/21 | 7/29/21 | 0.29323005 | 34.6982114 | 2.92981626 | 32.5853659 | -1.0305128 | 1.51636973 |
| DP.D.8.21.21 | DPMC1 | COOPMCAC1 | 6/8/21 | 8/19/21 | 0 | 36.2092131 | 2.07629194 | 32.0076516 | 0.04857882 | 1.15186789 |
| BDC.D.7.27.21 | UCDE | COOPDEEC1 | 6/5/21 | 7/27/21 | 0.48768 | 34.7081867 | 2.32900484 | 20.4458576 | -0.5772526 | 0.12069683 |
| PD.D.9.18.21 | CI200 | C2285 | 7/27/21 | 9/18/21 | 0.0395233 | 34.0172514 | 2.59678712 | 34.6449332 | 0.76791974 | -0.0186239 |
| WI.D.9.18.21 | CQ125 | D3583 | 7/29/21 | 9/18/21 | 0.2486511 | 34.0477588 | 2.61034694 | 38.7784026 | -0.8598018 | 1.16034868 |
| DP.D.9.18.21 | DPMC1 | COOPMCAC1 | 8/19/21 | 9/18/21 | 0.21166667 | 34.5156171 | 1.72249174 | 36.3112948 | 0.06495099 | 0.9728898 |
| BDC.D.9.20.21 | UCDE | COOPDEEC1 | 7/27/21 | 9/20/21 | 0.13854545 | 32.9690504 | 2.19504593 | 26.7910129 | -0.4308111 | 0.28229088 |
| PD.D.12.8.21 | CI200 | C2285 | 9/18/21 | 12/8/21 | 0.02522125 | 22.0988492 | 2.25398153 | 37.7321703 | 1.07625653 | -0.6142271 |
| WI.D.12.8.21 | CQ125 | D3583 | 9/18/21 | 12/8/21 | 0.00717957 | 21.413641 | 2.33039897 | 40.4497436 | 0.10867062 | 0.03284329 |
| DP.D.12.8.21 | DPMC1 | COOPMCAC1 | 9/18/21 | 12/8/21 | 0 | 23.3848234 | 1.64802729 | 30.438208 | 0.60341188 | 0.00521018 |
| BDC.D.12.8.21 | UCDE | COOPDEEC1 | 9/29/21 | 12/8/21 | 0.09012903 | 23.7779629 | 2.55024971 | 19.9816397 | 0.94124775 | 1.00408106 |

| **Sample ID** | **Average Shannon-Weiner Entropy** | **Average Shannon-Weiner Diversity** | **Average Species Richness** |
| --- | --- | --- | --- |
| PD.D.7.9.20 | 5.58651627 | 257.587506 | 268.39 |
| PD.D.8.14.20 | 5.49306021 | 251.884917 | 263.9 |
| PD.D.10.8.20 | 3.17969277 | 24.0374646 | 37.88 |
| PD.D.11.6.20 | 5.10144386 | 176.817695 | 226.54 |
| PD.D.7.27.21 | 2.85625398 | 15.1319115 | 54.76 |
| PD.D.9.18.21 | 5.16974897 | 203.481405 | 241.72 |
| PD.D.12.8.21 | 3.56770734 | 34.1414489 | 66.82 |
| BDC.D.7.9.20 | 4.04827087 | 56.8750242 | 97.25 |
| BDC.D.8.14.20 | 3.49191003 | 34.9647012 | 68.31 |
| BDC.D.10.8.20 | 3.35610049 | 31.71798 | 63.66 |
| BDC.D.11.6.20 | 5.55184743 | 262.400544 | 271.74 |
| BDC.D.7.27.21 | 3.50690192 | 31.019953 | 68.06 |
| BDC.D.9.20.21 | 5.43019555 | 229.814552 | 249.08 |
| BDC.D.12.8.21 | 4.18342686 | 67.095712 | 122.63 |
| DP.D.7.10.20 | 3.29487321 | 28.2201776 | 56.52 |
| DP.D.8.30.20 | 2.75125404 | 16.4169953 | 35.31 |
| DP.D.10.10.20 | 5.50130696 | 240.433636 | 259.06 |
| DP.D.11.5.20 | 5.45460927 | 242.601314 | 260.04 |
| DP.D.8.21.21 | 5.50859887 | 247.205155 | 262.46 |
| DP.D.9.18.21 | 3.43817222 | 34.1079388 | 74.6 |
| DP.D.12.8.21 | 5.29072436 | 204.054367 | 236 |
| WI.D.7.10.20 | 4.21581267 | 66.344652 | 118.37 |
| WI.D.8.30.20 | 2.61146746 | 16.5978292 | 33.71 |
| WI.D.10.10.20 | 2.60362997 | 13.5126999 | 22 |
| WI.D.11.5.20 | 5.06746513 | 173.0724 | 224.8 |
| WI.D.7.29.21 | 5.50692347 | 242.420872 | 259.22 |
| WI.D.9.18.21 | 2.21999821 | 10.4367424 | 39.95 |
| WI.D.12.8.21 | 4.81033473 | 118.698956 | 208.29 |

**Supplemental Table 5. Average Shannon-Weiner Diversity and Species Richness per Sample.** This table contains the average Shannon-Weiner entropy, average Shannon-Weiner diversity, and average species richness per sample.

| **Pairs** | **Sums of Squares** | **F value** | **R^2^** | **P value** | **P_adj_ value** |
| --- | --- | --- | --- | --- | --- |
| BDC vs DP | 15216.07 | 1.225157 | 0.09263834 | 0.1374 | 0.8244 |
| BDC vs PD | 24238.00 | 1.420882 | 0.10587098 | 0.1497 | 0.8982 |
| BDC vs WI | 12253.51 | 1.025153 | 0.07870566 | 0.3050 | 1.0000 |
| DP vs PD | 28382.01 | 1.523036 | 0.11262533 | 0.0902 | 0.5412 |
| DP vs WI | 13648.12 | 0.008762 | 0.07754479 | 0.3503 | 1.0000 |
| PD vs WI | 30062.35 | 1.654658 | 0.12117904 | 0.0717 | 0.4302 |

**Supplemental Table 6.** **Pairwise PERMANOVA Results Comparing Beta Diversity by Site.** This is a pairwise permutational multivariate analysis of variance (PERMANOVA) comparing the variance in beta diversity between sites.

| **Site(s)** | **Model** | **Model Variables** | **ordistep() AIC** | **VIF** | **Variance** | **F value** | **P value** |
| --- | --- | --- | --- | --- | --- | --- | --- |
| All | Developed STF + average wind speed + average accumulated precipitation (24 hrs) | Developed STF | 162.22 | 1.29 | 28.238 | 2.6477 | 0.0015 |
|  |  | average wind speed | 162.24 | 1.31 | 20.914 | 1.9609 | 0.0121 |
|  |  | average accumulated precipitation (24 hrs) | 162 | 1.02 | 22.500 | 2.1097 | 0.0317 |
| PD | average accumulated precipitation (24 hrs) + average wind speed | average accumulated precipitation (24 hrs) | 163.16 | 1.07 | 128.78 | 2.7279 | 0.001984 |
|  |  | average wind speed | 163.41 | 1.07 | 129.75 | 2.7485 | 0.011706 |
| BDC | average accumulated precipitation (24 hrs) + average meridional (north-south) component (v) | average accumulated precipitation (24 hrs) | 162.4 | 1.62 | 93.661 | 2.2868 | 0.04484 |
|  |  | average meridional (north-south) component (v) | 162.59 | 1.62 | 50.596 | 1.2354 | 0.29524 |
| DP | average accumulated precipitation (24 hrs) + Barren Land STF | average accumulated precipitation (24 hrs) | 163.46 | 1.06 | 65.890 | 2.3012 | 0.0131 |
|  |  | Barren Land STF | 163.56 | 1.06 | 46.379 | 1.6198 | 0.03413 |
| WI | average relative humidity + zonal (east-west) component (u) | average relative humidity | 163.92 | 1 | 80.367 | 2.5942 | 0.01964 |
|  |  | zonal (east-west) component (u) | 163 | 1 | 69.044 | 2.2287 | 0.01984 |

**Supplemental Table 7. Redundancy Analysis Results of Microbial Composition Across**

**and Within Sites.** These results show which environmental variables were

significant drivers of beta diversity across all four sites and within each site based

on a redundancy analysis (RDA).

| **Category** | **Gene** | **Model** | **Variables, Combinations, & Interactions** | **GLM Family** | **Adj R^2^ or McFadden’s R^2^** | **P value** |
| --- | --- | --- | --- | --- | --- | --- |
| **Sporulation** | *spoIVCA* | average accumulated precipitation (24 hrs) * average relative humidity | average accumulated precipitation (24 hrs) | Gaussian | 0.375 | 0.013 |
|  |  |  | average relative humidity |  |  | 0.016 |
|  |  |  | average accumulated precipitation (24 hrs) * average relative humidity |  |  | 0.068 |
|  | *spmA* | average accumulated precipitation (24 hrs) * average meridional (north-south) component (v) | average accumulated precipitation (24 hrs) | Poisson | 0.333 | 0.0009 |
|  |  |  | average meridional (north-south) component (v) |  |  | 0.089 |
|  |  |  | average accumulated precipitation (24 hrs) * average meridional (north-south) component (v) |  |  | 0.023 |
| **UV Radiation Resistance** | *lexA* | average meridional (north-south) component (v) | average meridional (north-south) component (v) | Poisson | 0.179 | 0.016 |
|  | *uvrA* | average meridional (north-south) component (v) + Open Water STF | average meridional (north-south) component (v) | Gaussian | 0.239 | 0.038 |
|  |  |  | Open Water STF |  |  | 0.019 |
|  |  |  | average meridional (north-south) component (v) + Open Water STF |  |  | 0.022 |
|  | *uvrB* | average meridional (north-south) component (v) + Open Water STF | average meridional (north-south) component (v) | Gaussian | 0.278 | 0.01 |
|  |  |  | Open Water STF |  |  | 0.032 |
|  |  |  | average meridional (north-south) component (v) + Open Water STF |  |  | 0.013 |
|  | *uvrC* | average meridional (north-south) component (v) + Open Water STF | average meridional (north-south) component (v) | Poisson | 0.327 | 0.022 |
|  |  |  | Open Water STF |  |  | 0.006 |
|  |  |  | average meridional (north-south) component (v) + Open Water STF |  |  |  |
| **Thermal Resistance** | *cspA* | average meridional (north-south) component (v) | average meridional (north-south) component (v) | Gaussian | 0.145 | 0.038 |
|  | *htpX* | Barren Land STF + Open Water STF | Barren Land STF | Gaussian | 0.402 | 0.0008 |
|  |  |  | Open Water STF |  |  | 0.001 |
|  |  |  | Barren Land STF + Open Water STF |  |  | 0.002 |
| **Osmotic Stress Resistance** | *osmY* | average accumulated precipitation (24 hrs) + average wind speed + average meridional (north-south) component (v) | average accumulated precipitation (24 hrs) | Poisson | 0.21 | 5.49e-7 |
|  |  |  | average wind speed |  |  | 0.0005 |
|  |  |  | average meridional (north-south) component (v) |  |  | 0.012 |
|  | *opuC* | average accumulated precipitation (24 hrs) * average wind speed | average accumulated precipitation (24 hrs) | Negative Binomial | 0.336 | 0.001 |
|  |  |  | average wind speed |  |  | 0.133 |
|  |  |  | average accumulated precipitation (24 hrs) * average wind speed |  |  | 0.017 |
| **LPS Synthesis/Modification** | *wecP* | average air temperature + zonal (east-west) component (u) | average air temperature | Gaussian | 0.442 | 0.016 |
|  |  |  | zonal (east-west) component (u) |  |  | 0.016 |
|  |  |  | average air temperature + zonal (east-west) component (u) |  |  | 0.00084 |
|  | *wbgP* | Salton Sea STF * Shrub STF | Salton Sea STF | Poisson | 0.327 | 1.29e-8 |
|  |  |  | Shrub STF |  |  | 0.00018 |
|  |  |  | Salton Sea STF * Shrub STF |  |  | 6.41e-5 |
| **Quorum Sensing** | *bisR* | average accumulated precipitation (24 hrs) + Crop Land STF | average accumulated precipitation (24 hrs) | Gaussian | 0.441 | 0.013 |
|  |  |  | Crop Land STF |  |  | 0.024 |
|  |  |  | average accumulated precipitation (24 hrs) + Crop Land STF |  |  | 0.0009 |
|  | *prgX* | average wind speed + Developed STF | average wind speed | Poisson | 0.334 | 0.0002 |
|  |  |  | Developed STF |  |  | 0.039 |
|  |  |  | average wind speed + Developed STF |  |  |  |

**Supplemental Table 8. Generalized Linear Models Results of Atmospheric Survival Adaptations (Genes) in the Metagenomes and Weather Variables.** This table contains the generalized linear models used to determine if climate variables significantly predicted the coverage of genes of interest across samples. This table is organized by functional categories of interest, the model for each gene within the category, the GLM family used, and the associated statistics for each model.

| **Bin.ID** | **Domain** | **Phylum** | **Class** | **Order** | **Family** | **Genus** | **Species** |
| --- | --- | --- | --- | --- | --- | --- | --- |
| WI.D.7.29.21.A.bin.8 | Bacteria | Actinobacteriota | Actinomycetia | Actinomycetales | Microbacteriaceae | Curtobacterium | Curtobacterium sp001705035 |
| WI.D.8.30.20.B.bin.17 | Bacteria | Actinobacteriota | Actinomycetia | Actinomycetales | Microbacteriaceae | Frondihabitans | Unknown |
| WI.D.8.30.20.B.bin.24 | Bacteria | Actinobacteriota | Actinomycetia | Actinomycetales | Microbacteriaceae | Frondihabitans | Unknown |
| WI.D.8.30.20.B.bin.6 | Bacteria | Actinobacteriota | Actinomycetia | Actinomycetales | Microbacteriaceae | Mycetocola_A | Unknown |
| DP.D.7.10.20.C.bin.62 | Bacteria | Actinobacteriota | Actinomycetia | Actinomycetales | Microbacteriaceae | Okibacterium | Unknown |
| DP.D.7.10.20.C.bin.54 | Bacteria | Actinobacteriota | Actinomycetia | Actinomycetales | Micrococcaceae | Pseudarthrobacter | Pseudarthrobacter phenanthrenivorans |
| BDC.D.11.6.20.A.bins.25 | Bacteria | Actinobacteriota | Actinomycetia | Mycobacteriales | Mycobacteriaceae | Corynebacterium | Corynebacterium sp012838715 |
| BDC.D.7.9.20.A.bin.10 | Bacteria | Actinobacteriota | Actinomycetia | Mycobacteriales | Mycobacteriaceae | Corynebacterium | Corynebacterium sp012838715 |
| BDC.D.9.20.21.B.bin.27 | Bacteria | Actinobacteriota | Actinomycetia | Mycobacteriales | Mycobacteriaceae | Corynebacterium | Corynebacterium sp012838715 |
| DP.D.11.5.20.A.bin.5 | Bacteria | Actinobacteriota | Actinomycetia | Mycobacteriales | Mycobacteriaceae | Corynebacterium | Corynebacterium sp012838715 |
| DP.D.12.8.21.B.bin.24 | Bacteria | Actinobacteriota | Actinomycetia | Mycobacteriales | Mycobacteriaceae | Corynebacterium | Corynebacterium sp012838715 |
| DP.D.8.21.21.A.bin.1 | Bacteria | Actinobacteriota | Actinomycetia | Mycobacteriales | Mycobacteriaceae | Corynebacterium | Corynebacterium sp012838715 |
| DP.D.8.30.20.A.bin.14 | Bacteria | Actinobacteriota | Actinomycetia | Mycobacteriales | Mycobacteriaceae | Corynebacterium | Corynebacterium sp012838715 |
| PD.D.8.14.20.B.bin.18 | Bacteria | Actinobacteriota | Actinomycetia | Mycobacteriales | Mycobacteriaceae | Corynebacterium | Corynebacterium sp012838715 |
| WI.D.11.5.20.A.bin.3 | Bacteria | Actinobacteriota | Actinomycetia | Mycobacteriales | Mycobacteriaceae | Corynebacterium | Corynebacterium sp012838715 |
| WI.D.12.8.21.A.bin.24 | Bacteria | Actinobacteriota | Actinomycetia | Mycobacteriales | Mycobacteriaceae | Corynebacterium | Corynebacterium sp012838715 |
| WI.D.7.10.20.A.bin.11 | Bacteria | Actinobacteriota | Actinomycetia | Mycobacteriales | Mycobacteriaceae | Corynebacterium | Corynebacterium sp012838715 |
| WI.D.7.29.21.A.bin.16 | Bacteria | Actinobacteriota | Actinomycetia | Mycobacteriales | Mycobacteriaceae | Corynebacterium | Corynebacterium sp012838715 |
| DP.D.12.8.21.B.bin.33 | Bacteria | Actinobacteriota | Actinomycetia | Propionibacteriales | Propionibacteriaceae | Cutibacterium | Cutibacterium acnes |
| WI.D.7.10.20.A.bin.8 | Bacteria | Bacteroidota | Bacteroidia | Bacteroidales | Tannerellaceae | Tannerella | Unknown |
| DP.D.7.10.20.C.bin.45 | Bacteria | Bacteroidota | Bacteroidia | Cytophagales | Hymenobacteraceae | Hymenobacter | Unknown |
| WI.D.8.30.20.B.bin.21 | Bacteria | Bacteroidota | Bacteroidia | Cytophagales | Spirosomaceae | Dyadobacter | Unknown |
| BDC.D.8.14.20.A.bin.42 | Bacteria | Bacteroidota | Bacteroidia | Cytophagales | Spirosomaceae | Spirosoma | Unknown |
| DP.D.7.10.20.C.bin.68 | Bacteria | Bacteroidota | Bacteroidia | Cytophagales | Spirosomaceae | Spirosoma | Unknown |
| BDC.D.8.14.20.A.bin.10 | Bacteria | Bacteroidota | Bacteroidia | Sphingobacteriales | Sphingobacteriaceae | Pedobacter | Unknown |
| BDC.D.9.20.21.B.bin.20 | Bacteria | Bacteroidota | Bacteroidia | Sphingobacteriales | Sphingobacteriaceae | Pedobacter | Unknown |
| WI.D.7.10.20.A.bin.15 | Bacteria | Desulfobacterota_I | Desulfovibrionia | Desulfovibrionales | Desulfovibrionaceae | Frigididesulfovibrio | Unknown |
| WI.D.9.18.21.A.bin.9 | Bacteria | Firmicutes | Bacilli | Bacillales | Bacillaceae_G | Ectobacillus | Unknown |
| BDC.D.7.27.21.A.bin.11 | Bacteria | Firmicutes | Bacilli | Bacillales | Bacillaceae_G | Ectobacillus | Unknown |
| BDC.D.7.27.21.A.bin.37 | Bacteria | Firmicutes | Bacilli | Bacillales | Bacillaceae_G | Ectobacillus | Unknown |
| PD.D.7.27.21.A.bin.27 | Bacteria | Firmicutes | Bacilli | Bacillales | Bacillaceae_G | Ectobacillus | Unknown |
| PD.D.7.27.21.A.bin.9 | Bacteria | Firmicutes | Bacilli | Bacillales | Bacillaceae_G | Ectobacillus | Unknown |
| WI.D.9.18.21.A.bin.21 | Bacteria | Firmicutes | Bacilli | Bacillales | Bacillaceae_G | Ectobacillus | Unknown |
| BDC.D.9.20.21.B.bin.1 | Bacteria | Firmicutes | Bacilli | Bacillales | Bacillaceae_H | Priestia | Priestia megaterium |
| WI.D.9.18.21.A.bin.6 | Bacteria | Firmicutes | Bacilli | Bacillales | Bacillaceae_H | Priestia | Priestia megaterium |
| BDC.D.12.8.21.A.bins.30 | Bacteria | Firmicutes | Bacilli | Bacillales_A | Planococcaceae | Planococcus | Unknown |
| PD.D.12.8.21.A.bin.18 | Bacteria | Firmicutes | Bacilli | Bacillales_A | Planococcaceae | Planococcus | Unknown |
| BDC.D.7.27.21.A.bin.3 | Bacteria | Firmicutes | Bacilli | Bacillales_B | DSM-18226 | Robertmurraya | Unknown |
| PD.D.7.27.21.A.bin.4 | Bacteria | Firmicutes | Bacilli | Bacillales_B | DSM-18226 | Robertmurraya | Unknown |
| WI.D.9.18.21.A.bin.2 | Bacteria | Firmicutes | Bacilli | Exiguobacterales | Exiguobacteraceae | Exiguobacterium_A | Exiguobacterium_A acetylicum |
| PD.D.7.27.21.A.bin.14 | Bacteria | Firmicutes | Bacilli | Paenibacillales | NBRC-103111 | Unknown | Unknown |
| BDC.D.11.6.20.A.bins.15 | Bacteria | Firmicutes | Bacilli | Staphylococcales | Salinicoccaceae | Salinicoccus | Salinicoccus roseus |
| BDC.D.7.9.20.A.bin.11 | Bacteria | Firmicutes | Bacilli | Staphylococcales | Salinicoccaceae | Salinicoccus | Salinicoccus roseus |
| BDC.D.9.20.21.B.bin.12 | Bacteria | Firmicutes | Bacilli | Staphylococcales | Salinicoccaceae | Salinicoccus | Salinicoccus roseus |
| DP.D.12.8.21.B.bin.23 | Bacteria | Firmicutes | Bacilli | Staphylococcales | Salinicoccaceae | Salinicoccus | Salinicoccus roseus |
| DP.D.8.21.21.A.bin.27 | Bacteria | Firmicutes | Bacilli | Staphylococcales | Salinicoccaceae | Salinicoccus | Salinicoccus roseus |
| DP.D.8.30.20.A.bin.5 | Bacteria | Firmicutes | Bacilli | Staphylococcales | Salinicoccaceae | Salinicoccus | Salinicoccus roseus |
| PD.D.8.14.20.B.bin.20 | Bacteria | Firmicutes | Bacilli | Staphylococcales | Salinicoccaceae | Salinicoccus | Salinicoccus roseus |
| WI.D.11.5.20.A.bin.19 | Bacteria | Firmicutes | Bacilli | Staphylococcales | Salinicoccaceae | Salinicoccus | Salinicoccus roseus |
| WI.D.12.8.21.A.bin.26 | Bacteria | Firmicutes | Bacilli | Staphylococcales | Salinicoccaceae | Salinicoccus | Salinicoccus roseus |
| WI.D.7.10.20.A.bin.5 | Bacteria | Firmicutes | Bacilli | Staphylococcales | Salinicoccaceae | Salinicoccus | Salinicoccus roseus |
| WI.D.7.29.21.A.bin.1 | Bacteria | Firmicutes | Bacilli | Staphylococcales | Salinicoccaceae | Salinicoccus | Salinicoccus roseus |
| WI.D.7.10.20.A.bin.1 | Bacteria | Firmicutes_A | Clostridia | Oscillospirales | Acutalibacteraceae | UBA945 | Unknown |
| BDC.D.12.8.21.A.bins.6 | Bacteria | Firmicutes_A | Clostridia | Oscillospirales | Ruminococcaceae | Unknown | Unknown |
| WI.D.11.5.20.A.bin.5 | Bacteria | Proteobacteria | Alphaproteobacteria | Acetobacterales | Acetobacteraceae | Unknown | Unknown |
| WI.D.12.8.21.A.bin.8 | Bacteria | Proteobacteria | Alphaproteobacteria | Acetobacterales | Acetobacteraceae | Unknown | Unknown |
| WI.D.7.29.21.A.bin.18 | Bacteria | Proteobacteria | Alphaproteobacteria | Acetobacterales | Acetobacteraceae | Unknown | Unknown |
| DP.D.7.10.20.C.bin.37 | Bacteria | Proteobacteria | Alphaproteobacteria | Acetobacterales | Acetobacteraceae | Belnapia | Unknown |
| DP.D.7.10.20.C.bin.73 | Bacteria | Proteobacteria | Alphaproteobacteria | Caulobacterales | Caulobacteraceae | Brevundimonas | Brevundimonas vesicularis |
| DP.D.12.8.21.B.bin.12 | Bacteria | Proteobacteria | Alphaproteobacteria | DSM-16000 | Inquilinaceae | Unknown | Unknown |
| DP.D.8.21.21.A.bin.14 | Bacteria | Proteobacteria | Alphaproteobacteria | DSM-16000 | Inquilinaceae | Unknown | Unknown |
| DP.D.7.10.20.C.bin.17 | Bacteria | Proteobacteria | Alphaproteobacteria | Rhizobiales | Devosiaceae | Devosia | Unknown |
| BDC.D.8.14.20.A.bin.3 | Bacteria | Proteobacteria | Alphaproteobacteria | Rhizobiales | Rhizobiaceae | Aureimonas_A | Unknown |
| BDC.D.11.6.20.A.bins.11 | Bacteria | Proteobacteria | Alphaproteobacteria | Rhizobiales_A | Rhizobiaceae_A | Bartonella | Bartonella sp016102285 |
| DP.D.9.8.21.B.bin.17 | Bacteria | Proteobacteria | Alphaproteobacteria | Rhizobiales_A | Rhizobiaceae_A | Bartonella | Bartonella sp016102285 |
| DP.D.7.10.20.C.bin.46 | Bacteria | Proteobacteria | Alphaproteobacteria | Rhodobacterales | Rhodobacteraceae | Cereibacter | Cereibacter changlensis |
| PD.D.7.27.21.A.bin.24 | Bacteria | Proteobacteria | Alphaproteobacteria | Sphingomonadales | Sphingomonadaceae | Unknown | Unknown |
| DP.D.7.10.20.C.bin.47 | Bacteria | Proteobacteria | Alphaproteobacteria | Sphingomonadales | Sphingomonadaceae | Novosphingobium | Unknown |
| PD.D.7.27.21.A.bin.23 | Bacteria | Proteobacteria | Gammaproteobacteria | Burkholderiales | Burkholderiaceae | Unknown | Unknown |
| WI.D.8.30.20.B.bin.19 | Bacteria | Proteobacteria | Gammaproteobacteria | Burkholderiales | Burkholderiaceae | Caballeronia | Unknown |
| BDC.D.7.27.21.A.bin.35 | Bacteria | Proteobacteria | Gammaproteobacteria | Burkholderiales | Burkholderiaceae | Noviherbaspirillum | Unknown |
| DP.D.9.8.21.B.bin.18 | Bacteria | Proteobacteria | Gammaproteobacteria | Burkholderiales | Burkholderiaceae | Noviherbaspirillum | Unknown |
| DP.D.7.10.20.C.bin.74 | Bacteria | Proteobacteria | Gammaproteobacteria | Burkholderiales | Burkholderiaceae | Pigmentiphaga | Unknown |
| BDC.D.9.20.21.B.bin.32 | Bacteria | Proteobacteria | Gammaproteobacteria | Burkholderiales | Burkholderiaceae | Pseudoduganella | Unknown |
| DP.D.7.10.20.C.bin.23 | Bacteria | Proteobacteria | Gammaproteobacteria | Burkholderiales | Burkholderiaceae | Pseudorhodoferax | Unknown |
| DP.D.7.10.20.C.bin.33 | Bacteria | Proteobacteria | Gammaproteobacteria | Burkholderiales | Burkholderiaceae | Pseudorhodoferax | Unknown |
| WI.D.11.5.20.A.bin.20 | Bacteria | Proteobacteria | Gammaproteobacteria | Burkholderiales | Burkholderiaceae | Robbsia | Unknown |
| WI.D.7.29.21.A.bin.6 | Bacteria | Proteobacteria | Gammaproteobacteria | CAIQBE01 | CAIQBE01 | Unknown | Unknown |
| WI.D.9.18.21.A.bin.10 | Bacteria | Proteobacteria | Gammaproteobacteria | Enterobacterales | Enterobacteriaceae | Enterobacter | Enterobacter kobei |
| DP.D.9.8.21.B.bin.1 | Bacteria | Proteobacteria | Gammaproteobacteria | Enterobacterales | Enterobacteriaceae | Gilliamella | Unknown |
| BDC.D.9.20.21.B.bin.4 | Bacteria | Proteobacteria | Gammaproteobacteria | Enterobacterales | Enterobacteriaceae | Gilliamella | Gilliamella apicola |
| BDC.D.7.9.20.A.bin.27 | Bacteria | Proteobacteria | Gammaproteobacteria | Enterobacterales_A | Enterobacteriaceae_A | Buchnera | Unknown |
| DP.D.12.8.21.B.bin.2 | Bacteria | Proteobacteria | Gammaproteobacteria | Enterobacterales_A | Enterobacteriaceae_A | Buchnera | Unknown |
| WI.D.11.5.20.A.bin.2 | Bacteria | Proteobacteria | Gammaproteobacteria | Enterobacterales_A | Enterobacteriaceae_A | Buchnera | Unknown |
| WI.D.11.5.20.A.bin.8 | Bacteria | Proteobacteria | Gammaproteobacteria | Enterobacterales_A | Enterobacteriaceae_A | Buchnera | Unknown |
| BDC.D.9.20.21.B.bin.30 | Bacteria | Proteobacteria | Gammaproteobacteria | Nitrococcales | Nitrococcaceae | Arhodomonas | Unknown |
| DP.D.12.8.21.B.bin.29 | Bacteria | Proteobacteria | Gammaproteobacteria | Nitrococcales | Nitrococcaceae | Arhodomonas | Unknown |
| DP.D.8.21.21.A.bin.20 | Bacteria | Proteobacteria | Gammaproteobacteria | Nitrococcales | Nitrococcaceae | Arhodomonas | Unknown |
| DP.D.12.8.21.B.bin.22 | Bacteria | Proteobacteria | Gammaproteobacteria | Pseudomonadales | Halomonadaceae | Zymobacter | Unknown |
| PD.D.7.27.21.A.bin.6 | Bacteria | Proteobacteria | Gammaproteobacteria | Pseudomonadales | Moraxellaceae | Acinetobacter | Acinetobacter baumannii |
| WI.D.9.18.21.A.bin.11 | Bacteria | Proteobacteria | Gammaproteobacteria | Pseudomonadales | Moraxellaceae | Acinetobacter | Acinetobacter baumannii |
| DP.D.9.8.21.B.bin.10 | Bacteria | Proteobacteria | Gammaproteobacteria | Pseudomonadales | Pseudomonadaceae | Entomomonas | Unknown |
| BDC.D.9.20.21.B.bin.23 | Bacteria | Proteobacteria | Gammaproteobacteria | Pseudomonadales | Pseudomonadaceae | Pseudomonas_E | Unknown |
| BDC.D.11.6.20.A.bins.20 | Bacteria | Proteobacteria | Gammaproteobacteria | Xanthomonadales | Xanthomonadaceae | Xanthomonas | Unknown |
| BDC.D.9.20.21.B.bin.29 | Bacteria | Proteobacteria | Gammaproteobacteria | Xanthomonadales | Xanthomonadaceae | Xanthomonas | Unknown |
| PD.D.11.6.20.B.bin.7 | Bacteria | Proteobacteria | Gammaproteobacteria | Xanthomonadales | Xanthomonadaceae | Xanthomonas | Unknown |
| PD.D.8.14.20.B.bin.24 | Bacteria | Proteobacteria | Gammaproteobacteria | Xanthomonadales | Xanthomonadaceae | Xanthomonas | Unknown |
| PD.D.9.18.21.C.bin.3 | Bacteria | Proteobacteria | Gammaproteobacteria | Xanthomonadales | Xanthomonadaceae | Xanthomonas | Unknown |
| WI.D.11.5.20.A.bin.24 | Bacteria | Spirochaetota | Spirochaetia | Borreliales | Borreliaceae | Unknown | Unknown |
| WI.D.12.8.21.A.bin.25 | Bacteria | Spirochaetota | Spirochaetia | Borreliales | Borreliaceae | Unknown | Unknown |
| WI.D.7.10.20.A.bin.13 | Bacteria | Spirochaetota | Spirochaetia | Borreliales | Borreliaceae | Unknown | Unknown |

**Supplemental Table 9. Taxonomic Annotation of Metagenome Assembled Genomes (MAGs).** This table contains the taxonomic annotation results from GTDB-tk of the high-quality MAGs found in this work.
